# Optimization of a photovoltaic/wind/battery energy-based microgrid in distribution network using machine learning and fuzzy multi-objective improved Kepler optimizer algorithms

**DOI:** 10.1038/s41598-024-64234-x

**Published:** 2024-06-10

**Authors:** Fude Duan, Mahdiyeh Eslami, Mohammad Khajehzadeh, Ali Basem, Dheyaa J. Jasim, Sivaprakasam Palani

**Affiliations:** 1 School of Intelligent Transportation, Nanjing Vocational College of Information Technology, Nanjing, 210000 Jiangsu China; 2 Department of Electrical Engineering, Kerman Branch, Islamic Azad University, Kerman, Iran; 3 Department of Civil Engineering, Anar Branch, Islamic Azad University, Anar, Iran; 4 Faculty of Engineering, Warith Al-Anbiyaa University, Karbala, 56001 Iraq; 5 Department of Petroleum Engineering, Al-Amarah University College, Maysan, Iraq; 6 College of Electrical and Mechanical Engineering, Addis Ababa Science and Technology University, 16417 Addis Ababa, Ethiopia

**Keywords:** Electrical and electronic engineering, Energy infrastructure

## Abstract

In this study, a fuzzy multi-objective framework is performed for optimization of a hybrid microgrid (HMG) including photovoltaic (PV) and wind energy sources linked with battery energy storage (PV/WT/BES) in a 33-bus distribution network to minimize the cost of energy losses, minimizing the voltage oscillations as well as power purchased minimization from the HMG incorporated forecasted data. The variables are microgrid optimal location and capacity of the HMG components in the network which are determined through a multi-objective improved Kepler optimization algorithm (MOIKOA) modeled by Kepler’s laws of planetary motion, piecewise linear chaotic map and using the FDMT. In this study, a machine learning approach using a multilayer perceptron artificial neural network (MLP-ANN) has been used to forecast solar radiation, wind speed, temperature, and load data. The optimization problem is implemented in three optimization scenarios based on real and forecasted data as well as the investigation of the battery's depth of discharge in the HMG optimization in the distribution network and its effects on the different objectives. The results including energy losses, voltage deviations, and purchased power from the HMG have been presented. Also, the MOIKOA superior capability is validated in comparison with the multi-objective conventional Kepler optimization algorithm, multi-objective particle swarm optimization, and multi-objective genetic algorithm in problem-solving. The findings are cleared that microgrid multi-objective optimization in the distribution network considering forecasted data based on the MLP-ANN causes an increase of 3.50%, 2.33%, and 1.98%, respectively, in annual energy losses, voltage deviation, and the purchased power cost from the HMG compared to the real data-based optimization. Also, the outcomes proved that increasing the battery depth of discharge causes the BES to have more participation in the HMG effectiveness on the distribution network objectives and affects the network energy losses and voltage deviation reduction.

## Introduction

### Motivation and background

Due to the significant rise in electrical usage over the past ten years, electricity distribution firms are now required to properly build and operate their networks in order to meet customer demands^1^. Thus, one of the primary ways to meet the demand from customers is through the incorporation of distributed generation resources (DG) into distribution networks^1^. Because of their benefits for clean energy and ecological sustainability, renewable energy sources like solar power and wind energy have received a lot of attention when it comes to distributed generation (DG) technology^2^. However, the distribution network's ability to function depends heavily on its placement of energy units. Moreover, there is still a problem with the time difference between the supply and demand for renewable energy, which can be resolved by incorporating energy storage^3^. Furthermore, since the best possible distribution of energy sources can minimize power losses and enhance system voltage features, dependability, power quality issues, etc., identifying and sizing energy sources in the distribution network is crucial and difficult^4^. Energy module placement can have benefits as well as drawbacks, of course. The greatest difficulty is figuring out where to put them and how big they should be in distribution networks. Conversely, the generated energy from wind and photovoltaic renewable energies is unpredictable because of their stochastic character^5,6^. This unpredictability has the potential to worsen voltage conditions in the distribution network and increase energy losses in conjunction with variations in production and load demand. On the contrary, when considering the efficiency of wind energy generation from the effects of climate and PV power from seasonal changes in irradiance, forecasting the power of these sources is very challenging^8^. The fluctuations in the power produced by renewable resources because of the intrinsic unpredictability of irradiance and wind speed have grown into an essential challenge when it comes to technical operation in the distribution network, so examining how these variables impact their uncertainty seems very important for network operators^7^. One way to tackle this difficulty is by using precise forecasting models to produce various renewable energy sources. To put it another way, to lessen the detrimental effects of uncertainty on the generation of these resources in the distribution networks, the allocation, and the renewable systems optimization in the network should be carried out while taking the uncertainty and meteorological data forecasting into consideration.

### Related works and gaps

To establish the best placement and capacity for renewable energy systems, several solutions have been presented for integration with distribution networks. The impact of assigning the DG units on power quality within the network is assessed in Dinh et al.^9^. The findings demonstrated that where and how big the DGs had a significant impact on the voltage-sag. In Arasteh et al.^10^, PV utilization in a distribution network is developed to lower costs and enhance the voltage conditions. The enhanced whale optimizer method is utilized to calculate the PV resource size. Single-objective DG sizing and placement are discussed in Ahmadi et al.^11^ to lower network losses when employing the symbiotic organisms search (SOS) technique. To minimize energy costs and CO_2_ emissions while taking into account energy that is not provided an attractive approach with the virus colony search (VCS) method is used in Hassan et al.^12^ for allocating the standalone energy system incorporated with PV and battery in the network. Using an improved salp swarm algorithm (ISSA), the best resource allocation and reconfiguring of the network are examined in Javad Aliabadi and Radmehr^13^ to reduce losses, enhance reliability, and take into account load demand, and generate resource uncertainty. The allocation of battery storage with PV connected to the network is studied in Wong et al.^14^ using the whale optimization algorithm (WOA) to reduce power loss. The results show that WOA effectively sizes and places the battery storage in the distribution network to minimize power loss; nevertheless, the PV system's uncertainty and changing demand behavior are not taken into account. Taking into account the commercial demand background, an enhanced bald eagle search algorithm is employed in Kamel et al.^15^ to minimize the power loss in the electrical networks. To increase network resilience, the methodology uses battery storage to charge from turbines under small demands. In Kumar and Injeti^16^, the butterfly optimization method is used to find a way to integrate biomass and BES with green distributed generation (DGs) in distribution networks. This optimizes the techno-economics for business distribution systems while taking network reconfiguration into account. Reducing voltage variation, annual economic loss, and power loss are the main objectives. The biomass and battery storage systems provide the WT and PV systems, respectively, to render non-controllable DGs, such as WT and PV, dispatchable. An improved sine cosine algorithm with the chaotic map is put forth in Selim et al.^17^ to reduce the power, mitigating voltage deviation, and voltage stability enhancement while optimizing the optimization of battery storage integrated with PV systems. To lower investment expenses and improve the voltage profile, a multi-verse optimization (MOMVO) is proposed in Ahmadi et al. ^11^ that incorporates both centralized and distributed generation. Phase two determines the size and location of the DGs, whereas the first phase optimizes the capacity and location of distributed generators. Nevertheless, effective probability distribution functions are not applied to describe the ambiguity of irradiance and wind, and the MOMVO algorithm's convergence properties are not assessed. A genetic algorithm is employed in Ahmadi et al. ^18^ to determine the best time to schedule BES in conjunction with PV systems to minimize energy loss, lessen reverse load flow in the distribution system, and reduce overvoltage. The optimizer's efficiency is not compared to alternative methods, and just one DG unit is allocated in all situations despite the uncertain nature of irradiance and dynamic load needs. Ref. ^19^ suggests that to improve distribution systems' dependability, PV and BES systems should be deployed simultaneously. A decomposition-based multi-objective evolution method is used to address the optimization issue, which is expressed as many goals and attempts to reduce energy losses, energy not provided, power quality, and load costs. The escaping bird search (EBS) method is used in Hamidan and Borousan ^20^ to propose a multi-objective, sequential assignment of two problems in hybrid DGs consisting of PVs, WTs, and battery storage in the network. This system can reduce overall losses and enhance power quality. In Moghaddam et al. ^21^, the weight factor approach is used to build a multi-criteria method to configure the networks using PV and WT renewable sources, while the moth-flame optimization (MFO) algorithm is used to improve reliability. The scheduling of an energy system with a PV and WT integrated with a system for storing batteries is examined in Jafar-Nowdeh et al. ^22^ in a distribution network to reduce energy losses, enhance reliability while accounting for uncertainties, and optimize the voltage profile. An enhanced escaping-bird search technique is used to achieve this goal. In Hadi Abdulwahid et al.^23^, Monte Carlo sampling (MCS) and particle swarm optimization (PSO) are employed to allocate WTs and reconfigure the network based on uncertainties in wind speed and irradiation. Utilizing an artificial hummingbird method that incorporates uncertainty and minimizes resource costs, emissions, and voltage variation, Wang et al.^24^ recommends an efficient allocation of renewable resources. A unique hybrid wind speed forecasting system determined by a deep learning neural network structure and the data area division technique is proposed in Ramadan et al.^25^. The wind speed data is broken down using complementary ensemble empirical mode decomposition in the data preprocessing module. Using machine learning methods, a comparative analysis of PV energy generation forecasts is offered in Liu et al. ^26^. The best predictive model for energy prediction, according to model efficiency, is the artificial neural network (ANN). For functioning day-ahead power forecasting that utilizes numerical temperature prediction, a head-to-head opposition of the physical, data-driven, and mixed approaches is carried out in Ledmaoui et al. ^27^. For optimizing the scale of an off-grid energy system, a data-driven approach is suggested in Mayer ^28^ that blends hybrid meta-heuristics and machine learning to anticipate weather patterns over the system's lifetime. A deep learning-driven allocating and battery storage operation and water electrolyzer is carried out in Abdullah et al. ^29^. To achieve this, a collection of real clean energy-curtailed data is thoroughly examined, and a prediction error and its PDF are found using deep learning forecasting techniques.

Several strategies are used to solve the issue in the context of the hybrid microgrid (HMG) energy systems optimization framework in the networks. These methods are offered to take into account four elements: projected data derived from machine learning algorithms; uncertainty; fuzzy multi-objective architecture; and battery energy storage. The following are some of the methods that have been used in the literature: (1) RES optimization in distribution networks without battery storage^9–14^, (2) RES optimization in distribution networks with battery storage^11,15–22^, (3) fuzzy multi-objective RES optimization^11,16,19–21^, and (4) RES optimization incorporating data forecasting^25–29^. As is evident, no research has been done on the hybrid PV/WT/BES microgrid systems optimization taking into account all four aspects according to the authors' knowledge so far.

### Contributions

The following is the paper's contributions considering the literature and gaps:

- For the hybrid PV/WT/BES microgrid system optimization in a distribution network, we built an innovative multi-objective improved mathematical framework instead of creating a single or bi-objective model that fails to accurately represent the actual scenario. A fuzzy decision-making technique (FDMT) is utilized to identify the final solution between the non-dominant options in the constructed multi-objective model, which takes three objectives into consideration: minimizing energy losses, minimizing voltage variations, and minimizing the cost of the HMG.
- The multilayer perceptron artificial neural network (MLP-ANN), a machine learning method, is employed in this work as an effective tool to forecast meteorological data and network load requirements in issue resolution. By utilizing this technology, network operators and renewable energy source operators may manage and integrate renewable energy sources into the grid with greater knowledge, creating an energy system that is more dependable and efficient. Technologies for storing energy are crucial, particularly those related to batteries and how deep they can be discharged (DOD) in HMGs.
- The literature study, however, reveals that there is a lack of attention paid to the effect of DOD on the optimization of HMG systems based on renewable resources combined with battery storage. Furthermore, the degree to which DOD modifications have improved the HMGs' efficiency with regard to distribution network losses and voltage circumstances has not been addressed.
- Nevertheless, no meta-heuristic method can be ideal for handling every optimization problem, according to the No Free Lunch (NFL) theorem^30^. As a result, it also has certain difficult issues, such as the propensity to enter local optimal and the excessive reliance on the quality of the original population. Because of this, the problem is solved in this study via a novel optimization method called the multi-objective improved Kepler optimization algorithm (MOIKOA), which was motivated by Kepler's laws of planetary motion^31^. To avoid becoming trapped in a local optimum, the piecewise linear chaotic map and FDMT are the foundations of the MOIKOA formulation.
- The objective is to create a fuzzy multi-objective structure that will enable the hybrid PV/WT linked with a BES (PV/WT/BES) microgrid system optimization in the network. This will be achieved via a multi-objective improved Kepler optimization algorithm (MOIKOA), which will take into account the effects of battery DOD changes and forecasted data based on MLP-ANN on microgrid optimization in the electrical networks.

### Paper organization

The problem constraints, multi-objective model, and HMG modeling are described in "Methodology" section. "Proposed optimizer" section describes the machine learning technique to data forecasting that uses a multilayer perceptron artificial neural network. In "Results and discussion" section, the suggested optimizer has been formulated. "Conclusion" section presents the simulation results, and Sect. 6 gives the research outcomes.

## Methodology

### Understudy microgrid

**Figure 1:**
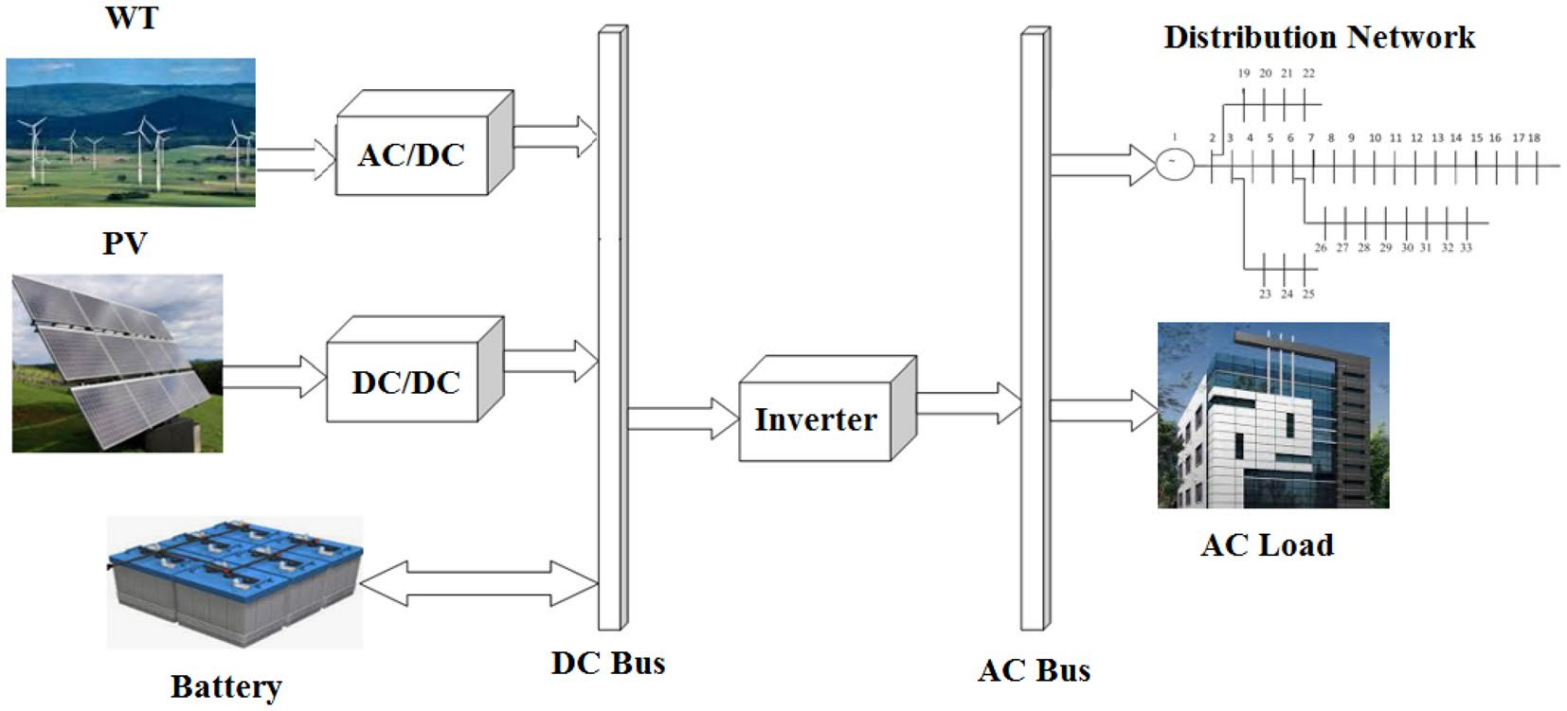
Schematic of the PV/WT/BES microgrid system.

The primary components of the proposed HMG system in this work are PV, WT, and battery energy storage (PV/WT/BES) according to Fig. 1. The batteries are depleted to fulfill the load with high reliability in the case of a system power outage. Batteries are used to store solar power surplus above load demands. After accepting DC power, the inverter is also responsible to supply the AC power to the AC demand. This technology is intended to first supply the HMG load and then introduce extra power into the network to enhance the network's operation.

### PV model

The following is a thorough model to calculate the PV array power considering the irradiance irradiated to the array's surface, the radiation's horizontal element, the surrounding temperature, and a few key technical features^32^:

1 $$P_{PV} \left( {S_{p} ,T_{c} } \right) = N_{pv} \times P_{r,pv} \times \frac{{S_{p} }}{{S_{STC} }} \times Et_{IE} \left( {S,T} \right)$$

2 $$S_{p} = \frac{S}{{{\text{sinh}}}} \times {\text{sin}}\left( {h + \beta } \right)$$

3 $$Et_{IE} \left( {S,T} \right) = 1 + k_{1} logS + k_{2} \left( {logS} \right)^{2} + T\left( {k_{3} + k_{4} logS + k_{5} \left( {logS} \right)^{2} } \right) + k_{6} T$$

4 $$T = T_{a} + \frac{{\left[ {\left( {NOCT - 20^{^\circ c} } \right).S_{p} } \right]}}{800} - T_{STC}$$

where ${P}_{r,pv}$ is rated PV power, ${Et}_{IE}$ is PV MPPT efficiency, $\beta$ is the PV surface angle versus the surface of horizontal, ${S}_{p}$ is the part of the solar radiation strength that is effectively radiated vertically on a plane that is inclined, $S$ displays the proportion of the radiation′s intensity to ${S}_{p}$ and nominal conditions (${S}_{STC}$ = 1000 W/m^2^)^32^, ${S}_{t}$ corresponds to the irradiation that the array's sloped surface emits, $h$ represents the irradiance head's angle, ${T}_{a}$ denotes the ambient temperature, $NOCT$ refers to the operation temperature normally (°C). ${k}_{1}-{k}_{6}$ are coefficient of ${Et}_{IE}\left(S,T\right)$ this is acquired by using actual data taken from one or more places to stabilize the model.

### Wind turbine model

The wind speed-based WT generating power model is a multi-parameter, nonlinear model. A combination of cut-in, cut-out, and nominal wind speed, the power generated by WT (${P}_{WT}$) is calculated by Szostok and Stanek^3^, Javad Aliabadi et al.^13^ and Moghaddam et al.^21^

5 $$P_{WT} = \left\{ {\begin{array}{*{20}l} {P_{rtd,WT} \times \left. {\left( {\frac{{V^{2} - V_{ci}^{2} }}{{V_{rtd}^{2} - V_{ci}^{2} }}} \right.} \right);} \hfill & {V_{ci} \le V \le V_{rtd} } \hfill \\ {P_{rtd,WT} ;} \hfill & {V_{rtd} \le V \le V_{co} } \hfill \\ 0 \hfill & {Otherwise} \hfill \\ \end{array} } \right.$$

where ${P}_{rtd,wt}$ is the PV nominal power, V is the wind speed, ${V}_{ci}$, ${V}_{rtd}$ and ${V}_{co}$ stand for cut-in, rated, and cut-out wind speeds (m/s). The wind data is recorded at a 40-m wind tower height, with an installation height of 15 m. The definition of wind speed under these circumstances is as follows Szostok and Stanek^3^, Javad Aliabadi et al.^13^ and Moghaddam et al.^21^:

6 $$V_{H} = V_{Href} \times \left. {\left( {\frac{{\text{H}}}{{H_{ref} }}} \right.} \right)^{\alpha }$$

where ${V}_{H}$ represents wind speed at height H, ${V}_{Href}$ represents wind speed at height ${H}_{ref}$, and α represents the surface's smoothness value, which ranges from 0.14 to 0.25^3,13,21^.

### Battery model

To maintain continuous load demand and offset PV power variations, battery storage has been employed. When there is excess power, the battery is charged with 40% of the power, with the remaining 40% being sent to the network. By draining the batteries, the load supply is kept at the appropriate level during a system power outage^11,14,15,18^:

#### Charging mode

When the electricity required for the load exceeds the PV production power $(P_{PV} \left( t \right) \times \eta_{DC/DC} > P_{LD} \left( t \right)/\eta_{Inv} )\; and\; E_{Batt} \left( {t - 1} \right) < E_{Batt}^{max}$, 40% of the extra power is fed into the batteries, which are charged to providing the full load. 60% of the surplus power is still delivered to the distribution system. The battery bank energy at t is defined by

7 $$E_{Batt} \left( t \right) = E_{Batt} \left( {t - 1} \right) \times \left( {1 - \sigma } \right) + \left[ {(P_{PV} \left( t \right) \times \eta_{DC/DC} ) - \frac{{(P_{LD} \left( t \right))}}{{\eta_{Inv} }}} \right] \times \eta_{ch} \times \Delta t$$

where σ represents the rate of self-discharge, ${P}_{LD}(t)$ indicated the demand at t, ${\eta }_{Inv}$ denotes efficiency of the inverter, Δt is the time step, and ${\eta }_{ch}$ denotes battery bank efficiency when charging.

#### Discharging mode

When the needed load is more than the PV generation power $(P_{PV} \left( t \right) \times \eta_{DC/DC} < P_{LD} \left( t \right)/\eta_{Inv} ) \;and \;E_{Batt} \left( {t - 1} \right) > E_{Batt}^{min}$, the system is now unable to serve the load due to a power shortfall. In this case, the battery bank is discharged to make up for the loss of system power. The battery energy at t can be expressed by

8 $$E_{Batt} \left( t \right) = E_{Batt} \left( {t - 1} \right) \times \left( {1 - \sigma } \right) - \left[ {\frac{{P_{LD} \left( t \right)}}{{\eta_{Inv} }} - (P_{PV} \left( t \right) \times \eta_{DC/DC} )} \right] \times \frac{\Delta t}{{\eta_{disch} }}$$

where ${\eta }_{disch}$ denotes discharging efficiency of the battery.

#### No charging and discharging mode

The demand is entirely provided and the saved energy remains constant when the PV output power and the demand are equal $(P_{PV} \left( t \right) \times \eta_{DC/DC} = P_{LD} \left( t \right)/\eta_{Inv} )$. The battery energy at t is modeled by

9 $$E_{Batt} \left( t \right) = E_{Batt} \left( {t - 1} \right)$$

### Objective function

The optimization of a PV/WT/BES microgrid system is implemented for energy losses, voltage deviations, and HMG power cos minimization.

### Minimization of the energy losses

The first aim in optimization of the PV/WT/BES microgrid system in the network is presented the energy losses cost (${C}_{loss}$) minimization which can be calculated by Hassan et al.^12^, Wong et al.^14^ and Saini, and Gidwani^19^

10 $$OF_{1} = \min (C_{loss} ) = \mathop \sum \limits_{{{\text{h}} = 1}}^{{\text{T}}} \mathop \sum \limits_{{{\text{l}} = 1}}^{{N_{l} }} {\text{P}}_{{{\text{loss}}}}^{{{\text{l}},{\text{h}}}} \times {\text{C}}_{{{\text{elect}}}}^{{\text{h}}}$$

where ${C}_{loss}$, ${\text{P}}_{\text{loss}}^{\text{l},\text{h}}$ and ${\text{C}}_{\text{elect}}^{\text{h}}$ denote the costs of losses, line *l* losses in hour *h*, and cost of electricity in hour *h*, respectively.

### Minimization of the voltage deviations

The second aim in the PV/WT/BES microgrid system optimization in the network is voltage deviations ($\text{VSD}$) minimization^33^ in the network buses which can be computed by

11 $$OF_{2} = \min ({\text{VSD}}) = \sqrt {\frac{1}{{N_{bus} }} \times \mathop \sum \limits_{i = 1}^{{N_{bus} }} \left( {v_{i} - v_{p} } \right)^{2} }$$

12 $$v_{p} = \frac{{\mathop \sum \nolimits_{i = 1}^{{N_{bus} }} v_{i} }}{{N_{bus} }}$$

where $\text{VSD}$ is the network voltage profile, *v*_*i*_ is voltage of ith bus, *v*_*p*_ is the buses mean voltage, and *N*_*bus*_ indicate bus numbers.

### Minimization of the HMG cost

The third aim in the PV/WT/BES microgrid system optimization in the network is minimizing the of total purchased power cost from the HMG (${C}_{HMG}$) for the network which is calculated by

13 $$OF_{3} = \min (C_{HMG} ) = \mathop \sum \limits_{h = 1}^{{\text{T}}} P_{Extra}^{h} \times C_{Extra}^{h} )$$

where ${P}_{Extra}^{h}$ is injected extra power to the network, and ${C}_{Extra}^{h}$ is cost of per kW for purchasing the extra power for the network.

### Multi-objective function

The problem of multi-objective optimization is formulated as follows in Lotfipour and Afrakhte^34^ and Nowdeh et al.^35^: It has many conflicting objective functions as well as equality and inequality constraints that must be simultaneously optimized.

14 $$MinF\left( x \right) = \left[ {f_{1} \left( x \right)f_{2} \left( x \right) \ldots f_{n} \left( x \right)} \right]^{T}$$

15 $$Subject\;\;to:\left\{ {\begin{array}{*{20}c} {g_{i} \left( x \right) < 0 i = 1,2, \ldots ,N_{ueq} } \\ {h_{i} \left( x \right) = 0 i = 1,2, \ldots ,N_{eq} } \\ \end{array} } \right.$$

where the number of objective functions is denoted by *n*, $F\left(x\right)$ is the vector of objective functions, and ${g}_{i}\left(x\right)$ and ${h}_{i}\left(x\right)$ are the constraints on inequality and equality. There are two possible solutions to a multi-objective optimization issue: x and y. One will either outweigh the other, or none of the other options will. Consequently, an answer x will win out more than an answer y if the next two conditions are met.

16 $${\forall j\in \left\{\text{1,2},\dots ,n\right\}, f}_{j}\left(x\right)\le {f}_{j}\left(y\right)$$

17 $${\exists k\in \left\{\text{1,2},\dots ,n\right\}, f}_{k}\left(x\right)<{f}_{k}\left(y\right)$$

Therefore, Pareto set solutions (PSSs) can be found via the non-dominant answers inside the search field. Ultimately, the earlier saved non-dominant answers contain the solution. To select the best answer among the best possible options, the fuzzy function is investigated using a membership function in which the exact variables number can be provided input. In this case, ${\mu }_{i}^{k}$, which has the following definition^34,35^, denotes for the objective function i value that corresponds to the PSS k:

18 $$\mu_{i}^{k} = \left\{ {\begin{array}{*{20}l} 1 \hfill & {f_{i} \le f_{i}^{min} } \hfill \\ {\frac{{f_{i}^{max} - f_{i} }}{{f_{i}^{max} - f_{i}^{min} }}} \hfill & {f_{i}^{max} < f_{i} < f_{i}^{min} } \hfill \\ {0 } \hfill & {f_{i} \ge f_{i}^{max} } \hfill \\ \end{array} } \right.$$

where the maximum and lower limits of the objective function I are, respectively, represented by ${f}_{i}^{max}$ and ${f}_{i}^{min}$. The recommended method of computing these values makes use of the optimization results for each objective function. Given that ${\mu }_{i}^{k}$ is a number between 0 and 1, a value of 0 denotes incompatibility between the answer and the operator's goals, while 1 denotes complete compliance.

The fuzzy decision-making process is designed to handle multiple conflicting objectives by assigning a membership function to each objective. This allows for a more flexible evaluation of trade-offs between objectives. For each solution in the Pareto front, the fuzzy decision-making process aggregates the fuzzy values of all objectives. This aggregation helps in ranking the solutions based on their overall performance across all objectives. Each objective is normalized, and membership functions are defined to convert the objective values into fuzzy values. This step ensures that different objectives are comparable and can be aggregated meaningfully. The fuzzy decision-making process identifies the solution with the highest aggregated membership value as the best compromise solution. This method helps in selecting a balanced solution that considers all objectives, rather than focusing on just one or a few. By employing the fuzzy decision-making process, the study ensures a comprehensive evaluation of all non-dominant solutions, leading to a final solution that best satisfies the multiple, often conflicting, objectives of minimizing energy losses, voltage oscillations, and power purchased from the grid, while optimizing the location and capacity of HMG components based on forecasted data.

#### Constraints

The items that follow limitations should apply to the objective function. The problem's restrictions are taken into account as follows Arasteh et al.^10^, Wong et al.^14^ and Saini, and Gidwani^19^:

#### Power balance

Throughout the whole study time, the network's production and consumption balance constraint is as outlined below:

19 $$\mathop \sum \limits_{h = 1}^{24} P_{slack}^{h} + P_{HMG}^{h} - P_{loss}^{h} - P_{D - DN}^{h} = 0$$

where ${P}_{slack}^{h}$, ${P}_{loss}^{h}$ and ${P}_{D-DN}^{h}$ refer to the power provided by the post, network losses and network demand at time *h*, and ${P}_{HMG}^{h}$ is transferred power for the network by the HMG at time h.

#### Renewable resources

The following restrictions should be met by the PV and wind urce sizes, with the PV panel's tilt with relation to the ground:

20 $$P_{PV}^{min} \le P_{PV} \le P_{PV}^{max}$$

21 $$0 \le \beta_{PV} \le 90$$

22 $$P_{WT}^{min} \le P_{WT} \le P_{WT}^{max}$$

where ${P}_{PV}^{min}$ and ${P}_{WT}^{min}$ are lower size of PVs and WTs sizes, ${P}_{PV}^{max}$ and ${P}_{WT}^{max}$ are upper size of PVs and WTs, and ${\beta }_{PV}$ is the panel surface angle with respect to the earth surface.

#### Battery SOC

The battery SOC should be satisfied by

23 $${{SOC}_{Batt}^{max}\left(1-DOD\right)=SOC}_{Batt}^{min}\le {SOC}_{Batt,h}\le {SOC}_{Batt}^{max}$$

where ${SOC}_{Batt}^{min}$ and ${SOC}_{Batt}^{max}$ are minimum and maximum values of battery SOC.

#### Bus voltage

Network bus voltages should fall between the following allowed ranges:

24 $${V}_{b}^{min}\le {V}_{b}\le {V}_{b}^{max}$$

where ${V}_{b}^{min}$ and ${V}_{b}^{max}$ are lower and upper limits of bus voltage.

#### Allowable current

The value of permitted current to flow via the network branches should satisfy the following constraint:

25 $$\left|{I}_{i}\right|\le \left|{I}_{i}^{max}\right| i=\text{1,2}, \dots , {N}_{l}$$

where ${I}_{i}^{max}$ is maximum allowable passed current from the network lines.

### Data forecasting based on the machine learning

**Figure 2:**
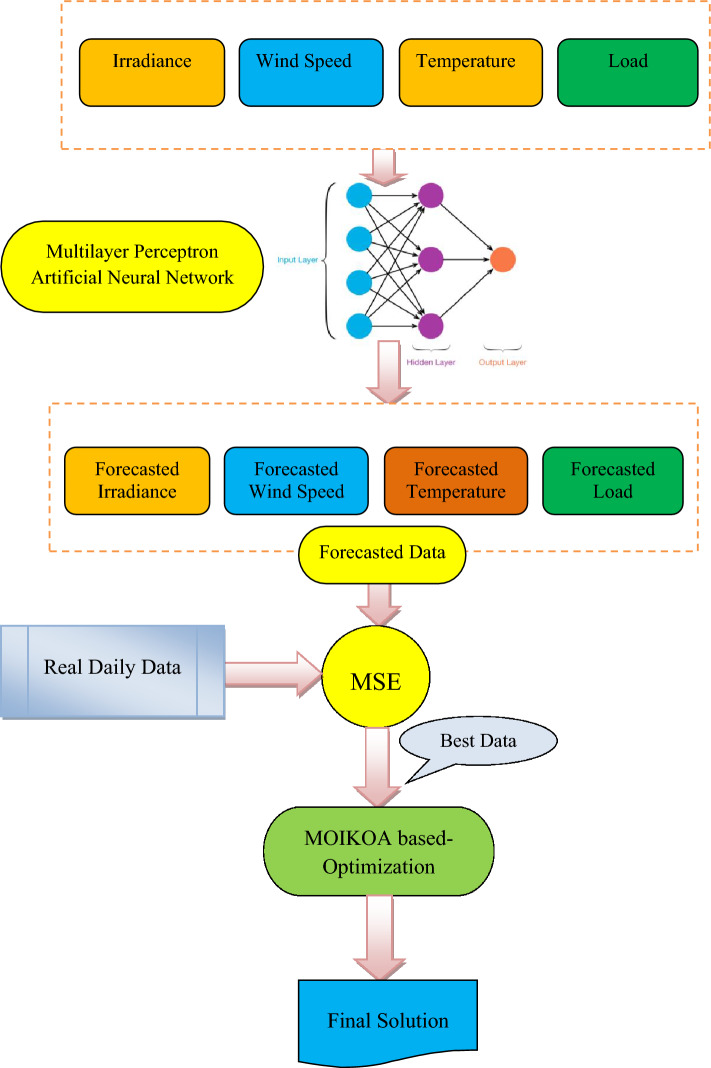
The MLP-ANN approach for forecasting the meteorological data and load demand.

In order to optimize the microgrid in the electrical network, the method of forecasting described utilizes a machine learning-based methodology that utilizes the multilayer perceptron artificial neural network (MLP-ANN) for daily load forecasting (Fig. 2). The machine learning-based method is applied to forecast temperature, wind speed, and radiation. Precisely predicting the electrical network's demand for load conditions and weather circumstances is essential for optimizing the energy microgrid within the network, especially when patterns of consumption get more intricate and dynamic. Nevertheless, forecasting weather errors leads to uncertainty, which deviates from microgrid optimization. The concentration of this research is on day-ahead microgrid optimization in the networks. To solve these difficulties, a machine learning-based technique for load demand and meteorological parameter predictions is employed.

### MPL-ANN algorithm

In this study, by forecasting future sampling utilizing previous data, the MLP-ANN is utilized to anticipate time-series values for load and weather parameters. This technique was chosen because, in contrast with additional complicated hybrid computations, it is easier to construct and has more technological maturation, thus being available to other academics who may be interested in using a similar approach. In recent research that focused on predicting certain parameters, it is usual practice to compare the predictions derived from this machine learning method in order to assess their efficacy^36–38^. Using the MLP-ANN technique, this study offers a multi-objective optimization of the microgrid in an electrical network, producing the most accurate predicted layout for each parameter (irradiance, wind speed, ambient temperature, and load demand). It is believed that a pattern that repeats itself once every 24 h is the ideal one. The mean squared error (MSE) measure is used for contrasting each anticipated pattern produced by the MPL-ANN method with the reality patterns. The patterns with the lowest MSE^36,37^ are selected for optimization. Figure 2 presents a summary of the recommended method and the pattern selection procedure.

This technique supports microgrid optimization by choosing exact patterns, which is in line with the present tendency of using machine learning techniques for predicting particular parameters.

26 $${\partial }_{Data}^{Best}=min\left\{MSE({\partial }_{Data-f}^{h},{\partial }_{Data-r}^{h})\right\},\forall \partial =\left\{S, V, {T}_{a}, Dmd\right\}$$

where for each parameter, ${\partial }_{Data}^{Best}$ represents the choice of the best-predicted pattern, ${\partial }_{Data-f}^{h}$ is predicted data at time t, ${\partial }_{Data-r}^{h}$ denotes real data at time t, $S$, $V$, ${T}_{a}$, and $Dmd$ refers to the radiation, wind speed, temperature and load demand, respectively. It computes the least mean square error (MSE) between the real pattern (${\partial }_{Data-f}^{h}$) and the predicted pattern (${\partial }_{Data-r}^{h}$).

### Predictive accuracy of the ANN

In this research, the predictive accuracy of the Artificial Neural Network (ANN) model is thoroughly assessed through various statistical parameters. These parameters include root mean square error (RMSE), mean squared error (MSE), standard deviation (STD), and linear regression (R). The focus is primarily on optimizing prediction performance, with Mean Squared Error (MSE) serving as the benchmark for identifying the most effective prediction pattern. ANN models learn patterns from historical data and adjust their parameters during training to minimize prediction errors. Their accuracy depends on factors like dataset size, quality, network architecture complexity, and optimization algorithms. ANN models leverage past data to make predictions on unseen data, offering powerful forecasting tools across various domains with their accuracy assessed through rigorous statistical analysis and validation techniques like cross-validation.

The suggested technique finds the best pattern for prediction via the Mean Squared Error (MSE).

27 $$R=\sqrt{\frac{\sum_{p=1}^{N}{({Y}_{p}-{X}_{p})}^{2}}{\sum_{p=1}^{N}{({X}_{p}-{Y}_{ave})}^{2}}}$$

28 $$MSE=\frac{1}{N}\sum_{p=1}^{N}{({Y}_{p}-{X}_{p})}^{2}$$

29 $$RMSE=\sqrt{\frac{1}{N}\sum_{p=1}^{N}{({Y}_{p}-{X}_{p})}^{2}}$$

According to recent studies Nguyen et al.^36^, the MLP-ANN is implemented for the prediction of time series data as it generates more satisfactory results. To analyze temporal information effectively, MLP-ANN requires a certain amount of memory. In ANN, memory is implemented through the use of time delays, and several different approaches have been devised to do this. In reality, these lags are employed to fine-tune ANN's learning process parameters. The layers that are input, hidden, and output are the three layers that make up this framework. Figure 2 shows single variation inputs, denoted as i(n-1),i(n-2)…i(n − p) match samples of earlier data, where O(n) represents the forecast's outcome value, while p indicates the prediction order.

## Proposed optimizer

### Overview of the Kepler optimization algorithm (KOA)

#### The KOA inspiration

Kepler's principles of planet motion are its main source of motivation. Kepler's laws state that the Earth's orbital acceleration, gravitational force and mass, and position of the planet are the four operators that influence a planet's course around the Sun. The mathematical framework of the suggested KOA was created using the operators. Because the planets in the KOA are in different conditions around the Sun at various times, the search space is more effectively investigated and utilized. Like other population-centered metaheuristic methods, KOA begins the search with an assortment of starting objects (the applicant solutions) that have probabilistic orbitals. At this point, each object's orbital location is initialized to be random. KOA evaluates the fitness of the initial set and repeats until the termination requirement is met. Since the word "time" is frequently applied in solar systems^39^ and cosmology, it is employed in place of "iteration" in the present investigation.

#### The KOA steps

The process of initializing process, defining the effect of gravity, computing an object's velocity, departing from the ideal location, updating objects' locations, maintaining distance from the sun, and exclusivity are some of the KOA stages that are covered in this part.

*Step 1 (the procedure under the initialization):* The formula listed below^31^ will be used in this procedure to randomly disperse planets are N, also known as the population size, in d-dimensions, which indicate the choice variables:

30 $$X_{i}^{j} = X_{i,low}^{j} + rand_{{\left[ {0,1} \right]}} \times \left( {X_{i,up}^{j} - X_{i,low}^{j} } \right),\left\{ {\begin{array}{*{20}c} {i = 1,2, \ldots ,N.} \\ {j = 1,2, \ldots ,d.} \\ \end{array} } \right.$$

where ${X}_{i,up}^{j}$ and ${X}_{i,low}^{j}$ denote the highest and lowest boundaries, correspondingly, of the j^th^ choice variable, and rand_[0,1]_ is an amount produced at random ranging from 0 to 1. Additionally, $X_{i}$ shows the i^th^ planet.

McDermott^31^ initializes the orbital eccentricity (e) for each ith object.

31 $${{e}_{i}=rand}_{[\text{0,1}]}, i=\text{1,2},\dots ,N.$$

where an arbitrary number created inside the interval [0, 1] is denoted by rand[0,1]. Lastly, McDermott^31^ initializes the orbital time (T) for each and every ith object.

32 $${T}_{i =}\left|r\right|, i=\text{1,2},\dots ,N.$$

where r is a random number that is produced using the typical distribution as the starting point.

*Step 2 (Determining the force of gravitational (F))*: The Sun is the central component of the radiation structure because it is the biggest item within the system and uses its gravity to direct the motion of the other objects. A planet's speed is determined by the Sun's gravitational pull. A planet's speed of orbit increases with increasing solar proximity, and vice versa. The universal rule of gravity, defined as follows, provides the Sun's gravitational attraction $X_{S}$ and each planet $X_{i}$^31^

33 $${F}_{gi}\left(t\right)={e}_{i}\times \mu \left(t\right)\times \frac{{\overline{M}}_{s}\times {\overline{m}}_{i}}{{\overline{R}}_{i}^{2}+\varepsilon }+{r}_{1}$$

where ${\overline{M}}_{s}$ and ${\overline{m}}_{i}$ are the normalized results of *M*_*S*_ and *m*_*i*_, which stand for the mass of of *X*_*S*_ and *X*_*i*_, $\mu$ is universal gravitational unchanged, e_i_ is the eccentricities of a planet's the orbit of the earth, which ranges from 0 to 1, r_1_ is generated at a random worth that ranges from 0 to 1 to add greater variability to the gravitation quantities during the optimization procedures; and ${\overline{R}}_{i}$ is the normalized amount of R_i_, and this is expressed as^31^.

34 $${R}_{i}\left(t\right)={\Vert {X}_{S}\left(t\right)-{X}_{i}\left(t\right)\Vert }_{2}=\sqrt{\sum_{j=1}^{d}{({X}_{Sj}\left(t\right)-{X}_{ij}\left(t\right))}^{2}}$$

In this case, the Euclidean space across the dimensions of ${X}_{S}$ and ${X}_{i}$ is represented by ${\Vert {X}_{S}\left(t\right)-{X}_{i}\left(t\right)\Vert }_{2}$. Applying the fitness evaluation, the Sun's mass and object i at time t is computed^31^

35 $${M}_{s}=\frac{{fit}_{s}\left(t\right)-worst\left(t\right)}{\sum_{k=1}^{N}({fit}_{k}\left(t\right)-worst(t)}$$

36 $${m}_{i}=\frac{{fit}_{i}\left(t\right)-worst\left(t\right)}{\sum_{k=1}^{N}({fit}_{i}\left(t\right)-worst(t)}$$

where

37 $${fit}_{s}\left(t\right)=best\left(t\right)=\text{min}{fit}_{k}\left(t\right),\text{ k}\in \text{1,2},\dots ,\text{N}.$$

38 $$worst\left(t\right)=\text{max}{fit}_{k}\left(t\right),\text{ k}\in \text{1,2},\dots ,\text{N}.$$

where r_2_ is a number produced at random between 0 and 1 that is used to separate the mass numbers of different planets. $\mu (t)$ is a function to control the search precision that progressively diminishes with time (t) and has the following definition^31^:

39 $$\mu \left(t\right)={\mu }_{0}\times \text{exp}(-\gamma \frac{t}{{T}_{max}})$$

where t and ${T}_{max}$ are presented iteration number and the greatest iterations number, respectively, while $\gamma$ is a fixed number and ${\mu }_{0}$ is a starting amount.

*Step 3 (Computing the object’ speed)*: A velocity of object is determined by its location in relation to the sun. To put it another way, a planet's velocity rises in response to the sun's strong gravitational pull when it approaches the sun and vice versa. This kind of action can be expressed scientifically as a dual solution. In the first step, the distance between the existing answer and an arbitrarily chosen solution is multiplied to find the speeds of planets near the Sun. In order to help maintain the velocity of the planet during the optimization procedure and prevent becoming caught in regional minima, a second step that depends on the separation among the upper and lower boundaries of the challenge is considered at the initial fold. The following is how this pattern of conduct is expressed^31^:

40 $$V_{i} \left( t \right) = \left\{ \begin{gathered} \ell \times \left( {2r_{4} \vec{X}_{i} - \vec{X}_{b} } \right) + \ddot{\ell } \times \left( {\vec{X}_{a} - \vec{X}_{b} } \right) + \left( {1 - R_{i - norm} \left( t \right)} \right) \times {\mathcal{F}} \times \vec{U}_{1} \times \vec{r}_{5} \times \left( {\vec{X}_{i,up} - \vec{X}_{i,low} } \right),\;\;\; if R_{i - norm} \left( t \right) \le 0.5 \hfill \\ \quad r_{4} \times {\mathcal{L}} \times \left( {\vec{X}_{a} - \vec{X}_{i} } \right) + \left( {1 - R_{i - norm} \left( t \right)} \right) \times {\mathcal{F}} \times U_{2} \times \vec{r}_{5} \times \left( {r_{3} \vec{X}_{i,up} - \vec{X}_{i,low} } \right),\;\; Else \hfill \\ \end{gathered} \right.$$

41 $${\ell}=\overrightarrow{U}\times \mathcal{M}\times \mathcal{L}$$

42 $$\mathcal{L}={\left[\mu \left(t\right)\times ({M}_{S}+{m}_{i})\left|\frac{2}{{R}_{i}\left(t\right)+\varepsilon }-\frac{1}{{a}_{i}\left(t\right)+\varepsilon }\right|\right]}^{0.5}$$

43 $$\mathcal{M}={r}_{3}\times \left(1-{r}_{4}\right)+{r}_{4}$$

44 $$\overrightarrow{U}=\left\{\begin{array}{c}0 {\overrightarrow{r}}_{5}\le {\overrightarrow{r}}_{6} \\ 1 \, \, \, \, \, \, \, \, \, \, Else\end{array}\right.$$

45 $$\mathcal{F}=\left\{\begin{array}{c}1, if {r}_{4}\le 0.5 \\ -1 \, \, \, \, \, \, \, \, \, \, \, \, Else\end{array}\right.$$

46 $$\ddot{{\ell}}=(1-\overrightarrow{U})\times \overrightarrow{\mathcal{M}}\times \mathcal{L}$$

47 $$\overrightarrow{\mathcal{M}}=({r}_{3}\times \left(1-{\overrightarrow{r}}_{5}\right)+{\overrightarrow{r}}_{5})$$

48 $${\overrightarrow{U}}_{1}=\left\{\begin{array}{c}0 {\overrightarrow{r}}_{5}\le {r}_{4} \\ 1 \, \, \, \, \, \, \, \, \, \, Else\end{array}\right.$$

49 $${U}_{2}=\left\{\begin{array}{c}0 {r}_{3}\le {r}_{4} \\ 1 \, \, \, \, \, \,Else\end{array}\right.$$

where r_3_ and r_4_ are arbitrarily produced numerical numbers at range [0, 1], ${\overrightarrow{r}}_{5}$ and ${\overrightarrow{r}}_{6}$ are a couple of vectors containing arbitrary numbers from 0 to 1, ${V}_{i}\left(t\right)$ denotes the acceleration of object i, and ${\overrightarrow{X}}_{i}$ indicates object i. ${\overrightarrow{X}}_{a}$ and ${\overrightarrow{X}}_{b}$ are randomly chosen solutions taken from the population, M_S_ and mi denote ${X}_{S}$ and ${X}_{i}$'s respective masses; $\mu \left(t\right)$ is constant of the universal gravitational^31^. ɛ is a tiny quantity that eliminates a division by zero error.

50 $${a}_{i}\left(t\right)={{r}_{3}\times \left[{T}_{i}^{2}\times \frac{\mu \left(t\right)\times ({M}_{S}+{m}_{i})}{4{\pi }^{2}}\right]}^{1/3}$$

where T_i_ stands for object i's orbital period.

${R}_{i-norm}\left(t\right)$ is Euclidian normal distance among ${X}_{S}$ and ${X}_{i}$, and it is determined by

51 $${R}_{i-norm}\left(t\right)=\frac{{R}_{i}\left(t\right)-\text{min}(R\left(t\right))}{\text{max}\left(R\left(t\right)\right)-\text{min}(R\left(t\right))}$$

If ${R}_{i-norm}\left(t\right)\le 0.5$, the object is near the Sun and will accelerate to avoid falling in its direction due to the powerful attraction of the Sun.

*Step 4 (Leaving the local optimal)*: Most objects in the radiation system's axes of rotation in a counterclockwise direction around the Sun, but some objects also rotate in a circular motion direction. This characteristic is exploited by the recommended method to escape local optimum areas. By considering a flag $\mathcal{F}$ to alter the search orientation and increase the likelihood of agents successfully exploring the search space, the suggested KOA mimics this pattern of action.

**Figure 3:**
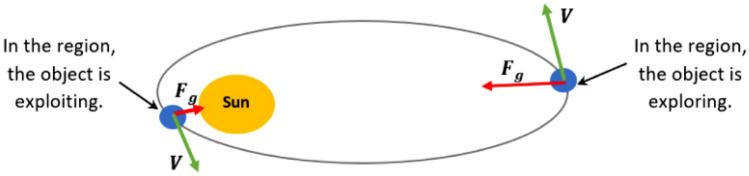
Locations for discovery and exploitation in the area of searches^31^.

*Step 5 (Updating objects’ positions)*: Objects rotate in a direction that brings them closer to the Sun for a while before turning aside. The two main stages of the suggested algorithm—the discovery and utilization phases—simulate this tendency. KOA looks for new locations near the best options when utilizing solutions closer to the Sun to identify new items to explore and discover new solutions. The areas of exploration and harvesting surrounding the Sun are depicted in Fig. 3. The objects' distance from the Sun during the exploration stage suggests that the recommended technique more effectively covers the whole search region. Following the preceding procedures, McDermott^31^ updates the position of every object that is far from the Sun.

52 $${\overrightarrow{X}}_{i}\left(t+1\right)={\overrightarrow{X}}_{i}\left(t\right)+\mathcal{F}\times {\overrightarrow{V}}_{i}\left(t\right)+{({\mathbb{F}}}_{{g}_{i}}+\left|r\right|)\times \overrightarrow{U}\times ({\overrightarrow{X}}_{S}\left(t\right)-{\overrightarrow{X}}_{i}\left(t\right))$$

where $\overrightarrow {X}_{i} (t + 1)$ is the most favorable location of the Sun found to date, $\overrightarrow {V}_{i} (t)$ denotes the object i new position at time t + 1, $X_{S} (t)$ is the speed of object i required for reaching the new position, and $\Im$ is employed as a flag to modify the orientation of the search.

*Step 6 (Updating distance with the Sun)*: KOA prioritizes exploitation actor optimization while planets are close to the Sun and optimize the exploration operator when planets are far from the Sun. The regulatory parameter h's value, which varies steadily with time, determines the application of these principles. If this quantity is modest, to exploit, the exploitation operator is employed for the areas surrounding the best-so-far answer if the distance between the Sun and planets is short. On the other hand, when this is great, an exploring operator is used to increase the planet's orbital dispersion from the Sun. As stated in Algorithm 1, this idea is randomly swapped out to enhance KOA's research and extraction operators even further. The following is a description of this principle's model^31^:

53 $${\overrightarrow{X}}_{i}\left(t+1\right)={\overrightarrow{X}}_{i}\left(t\right)\times {\overrightarrow{U}}_{1}+\left(1-{\overrightarrow{U}}_{1}\right)\times (\frac{{\overrightarrow{X}}_{i}\left(t\right)+{\overrightarrow{X}}_{S}+{\overrightarrow{X}}_{a}\left(t\right)}{3}+h\times (\frac{{\overrightarrow{X}}_{i}\left(t\right)+{\overrightarrow{X}}_{S}+{\overrightarrow{X}}_{a}\left(t\right)}{3}-{\overrightarrow{X}}_{b}\left(t\right)))$$

As explained following McDermott^31^, ℎ denotes an adaptive factor that controls the separation across the Sun and the present-day planet.

54 $$h=\frac{1}{{e}^{\eta r}}$$

where $\mu$ is a linearly declining factor from 1 to − 2, as stated following McDermott^31^, and r is a number created statistically based on a normal distribution:

55 $$\eta =\left({a}_{2}-1\right)\times {r}_{4}+1$$

where a_2_ is a cyclic regulating parameter that gradually falls for periods during the entire optimization procedure from − 1 to − 2 in the following definition of the procedure of optimization as a whole^31^ for $\overline{T}$ cycles:

56 $${a}_{2}=-1-1\times \frac{t\text{\%}\frac{{T}_{max}}{\overline{T}}}{\frac{{T}_{max}}{\overline{T}}}$$

*Step 7 (Elitism)*: To ensure the planets are positioned as optimally as possible and the Sun, this stage employs an elite method. This procedure is summarized in McDermott^31^.

57 $$\vec{X}_{i,new} \left( {t + 1} \right) = \left\{ {\begin{array}{*{20}l} {\vec{X}_{i} \left( {t + 1} \right),} \hfill & {if\;\;f\left( {\vec{X}_{i} \left( {t + 1} \right)} \right) \le f\left( {\vec{X}_{i} \left( t \right)} \right)} \hfill \\ {\vec{X}_{i} \left( t \right) } \hfill & {Else} \hfill \\ \end{array} } \right.$$

### Overview of the improved KOA (IKOA)

To improve algorithm search efficiency and prevent the KOA from becoming trapped in local optimization, the chaotic sequences are integrated into the KOA. For increasing the search size, the piecewise linear chaotic map (PLCM)^40^ is utilized to produce the chaotic sequence. KOA utilizes the PLCM to generate chaotic sequences. PLCM is known for its ability to generate chaotic behavior with a controllable degree of randomness. By leveraging PLCM, KOA can increase the search size, enabling it to explore a larger solution space efficiently. This feature helps KOA to avoid getting trapped in local optima and facilitates the discovery of diverse and potentially better solutions.

The starting point of the chaotic sequence element is expressed by

58 $${Z}_{0}=rand(\text{0,1})$$

The following variables of the PLCM-based chaotic sequence are specified theoretically as

59 $$Z_{k + 1} = \left\{ {\begin{array}{*{20}l} {\frac{{Z_{k} }}{p}} \hfill & {Z_{k} \in \left( {0,p} \right)} \hfill \\ {\frac{{1 - Z_{k} }}{1 - p}} \hfill & {Z_{k} \in \left( {p,1} \right)} \hfill \\ \end{array} } \right.$$

To help the current search group members find more effective approaches more quickly, the chaotic local search is included. The present search group member generates a new solution using the corresponding equation:

60 $${X}_{ik,new}={X}_{ik}+r(2{Z}_{k}-1)$$

where Z_k_ indicates the chaotic parameter at the kth iteration, X_ik_ represents the ith member location within the search group at the kth each iteration, and X_ik,new_ refers to the new solution location produced by chaotic local exploration at the kth iteration.

First, as follows, the chaotic searching radius (*r*) is established and adjusted in subsequent iterations:

61 $$r=\frac{({X}_{max}-{X}_{min})}{2}$$

62 $${r}^{k+1}=rand(\text{0,1})\times {r}^{k}$$

If *X*_*ik,new*_ OF quantity is higher than *X*_*ik*_'s, it will replace *X*_*ik*_ in the search group. Until the greatest number of iterations (*K*) of the chaotic neighborhood hunt is obtained, the chaotic localized search is carried out.

### Implementing the IKOA

**Figure 4:**
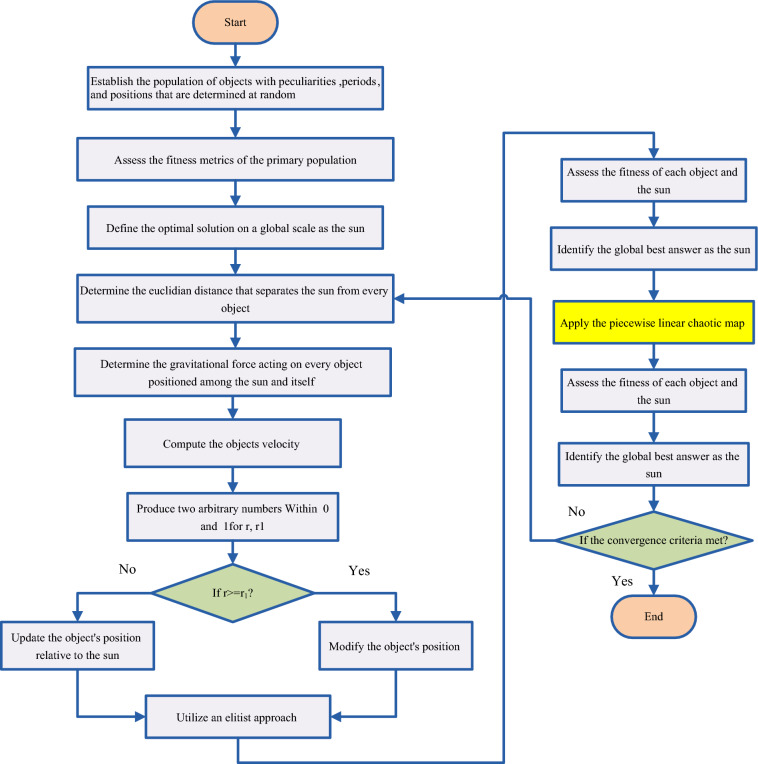
Flowchart of the IKOA.

The phases of multi-objective optimization of the energy microgrid in the network are provided utilizing the MOIKOA and the idea of the FDMT. The recommended methodology's flowchart is displayed in Fig. 4.

*Step 1:* The program has been implemented using data from the network buses and lines, microgrid device data, including PV and wind sources and batteries, meteorological data, and network load needs. The program is additionally considered he general (population size, maximum iteration, and number of separate executions) and adjusting parameters of the optimizer.

*Step 2:* For each algorithm's population, the decision variables (size of each piece of equipment and the placement of the HMG installation within the network) are chosen at random.

*Step 3:* Every set of chosen variables for the algorithm's population has its objective function Eqs. (14)–(18) determined.

*Step 4:* Once the non-dominated solutions have been identified, they are sorted and stored in the archive.

*Step 5:* The most effective member is chosen from the archive of non-dominant solutions.

*Step 6:* Based on Eq. (52) the population of the algorithm is updated.

*Step 7:* The solutions that are non-dominated are separated and archived, and the objective function for the population that was modified in Step 6 is computed. Substitute the optimal population member identified in step 5 with the best member found in the archive.

*Step 8:* Using Eq. (53), the algorithm's population is updated.

*Step 9:* The non-dominated answers are separated and stored following the algorithm's population update in step 8. The best member identified in step 7 is replaced based on the best population member from the non-dominated solution archive.

*Step 10:* Using the piecewise linear chaotic map, Eqs. (58)–(62), the algorithm's population is updated.

*Step 11:* The non-dominated answers are separated and archived, and the objective function for the population that was modified in Step 10 is computed. Step 9's best member should be used in place of the best population member found in the archive.

*Step 12:* Archive current non-dominant solutions and eliminate archived dominant solutions.

*Step 13:* Convergence circumstance assessment. Proceed to step 14 if the convergence is obtained through executing the greatest iteration number; otherwise, proceed to step 6.

*Step 14:* Terminate the program and preserve the best interactive solution.

MOIKOA improves decision-making for fuzzy solutions by leveraging its enhanced exploration capabilities. Fuzzy solutions often arise in multi-objective optimization problems where the objectives conflict with each other. By employing chaotic sequences and expanding the search space, MOIKOA can better explore the trade-off between conflicting objectives, leading to the identification of Pareto-optimal solutions that provide a balanced compromise among competing objectives. This ability to handle fuzzy solutions distinguishes MOIKOA from KOA, making it more suitable for complex optimization problems with multiple conflicting objectives.

## Results and discussion

**Figure 5:**
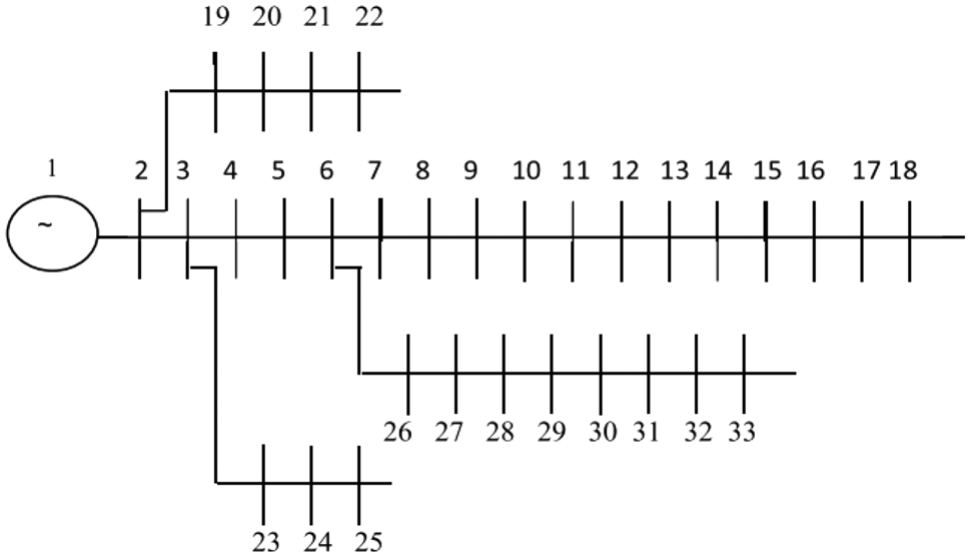
The schematic of 33-bus network.

The outcomes of fuzzy multi-objective optimization of a PV/WT/BES microgrid system are given in the network via the MOIKOA considering uncertainty and forecasted data based on the MLP-ANN. The suggested method is performed on a 33-bus network according to Fig. 5. The bus and line data of this network is taken from^41^. The active and reactive demand of the 33-bus network is 3.72 MW and 2.3 MVAr^41^. Data of microgrid components cost and technical data of microgrid components are given in Tables 1 and 2, respectively.

**Table 1:** Data of microgrid components cost^10,13,32^.

Item/device	WT	PV array	Battery bank
Investment cost ($)	3000	2300	213
Cost of O&M ($)	90	23	7
Life span (yr)	20	20	5
Size	1 kW	1 kW	1 kAh

**Table 2:** Technical data of microgrid components^10,13,32^.

PV	Value
Rated temperature (C)	25
Rated irradiance (w/m2)	103
WT	Value
${V}_{ci}$(m/s)	3
${V}_{co}$(m/s)	9
${V}_{rtd}$(m/s)	20
Battery	Value
Voltage (V)	12
Lower size (kWh)	20% of maximum size
DOD	0.8
Efficiency of charge and discharge	0.9

To evaluate proposed methodology the below scenarios are as follows:

*Scenario#1:* Fuzzy multi-objective optimizing the PV/WT/BES system with real data.

*Scenario#2:* Fuzzy multi-objective optimizing the PV/WT/BES system with forecasted data.

*Scenario#3:* Fuzzy multi-objective optimizing the PV/WT/BES system with forecasted data incorporating the DOD variations effect using the MOIKOA.

**Figure 6:**
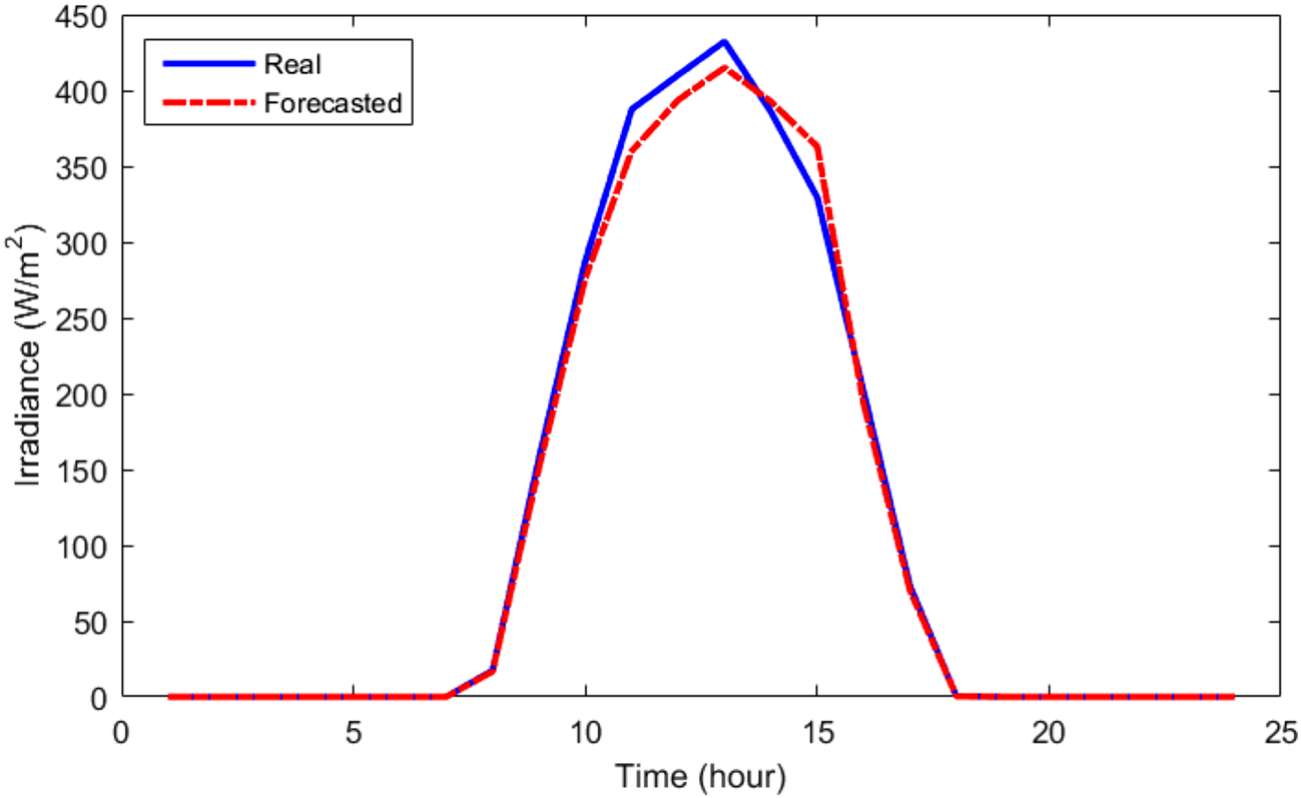
The real and forecasted irradiance during a day.

**Figure 7:**
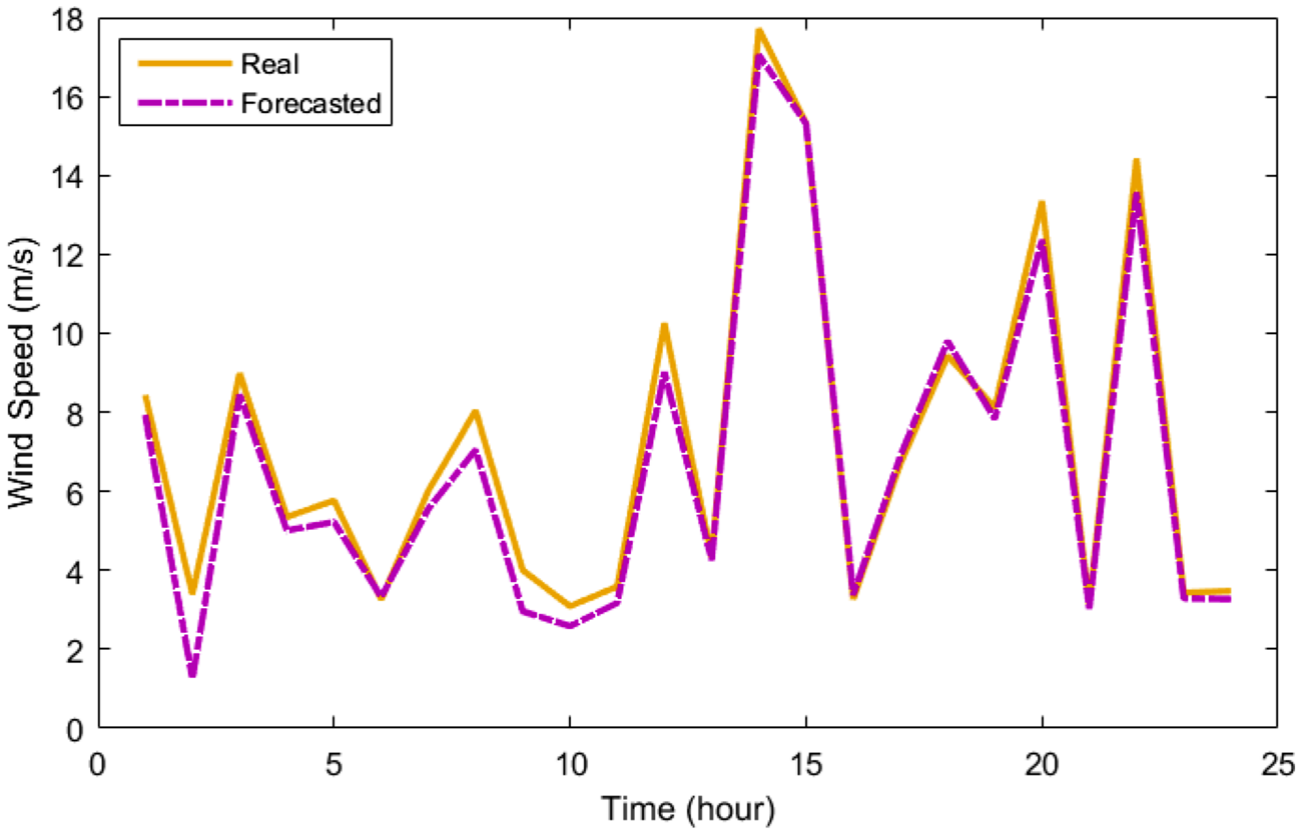
The real and forecasted wind speed during a day.

**Figure 8:**
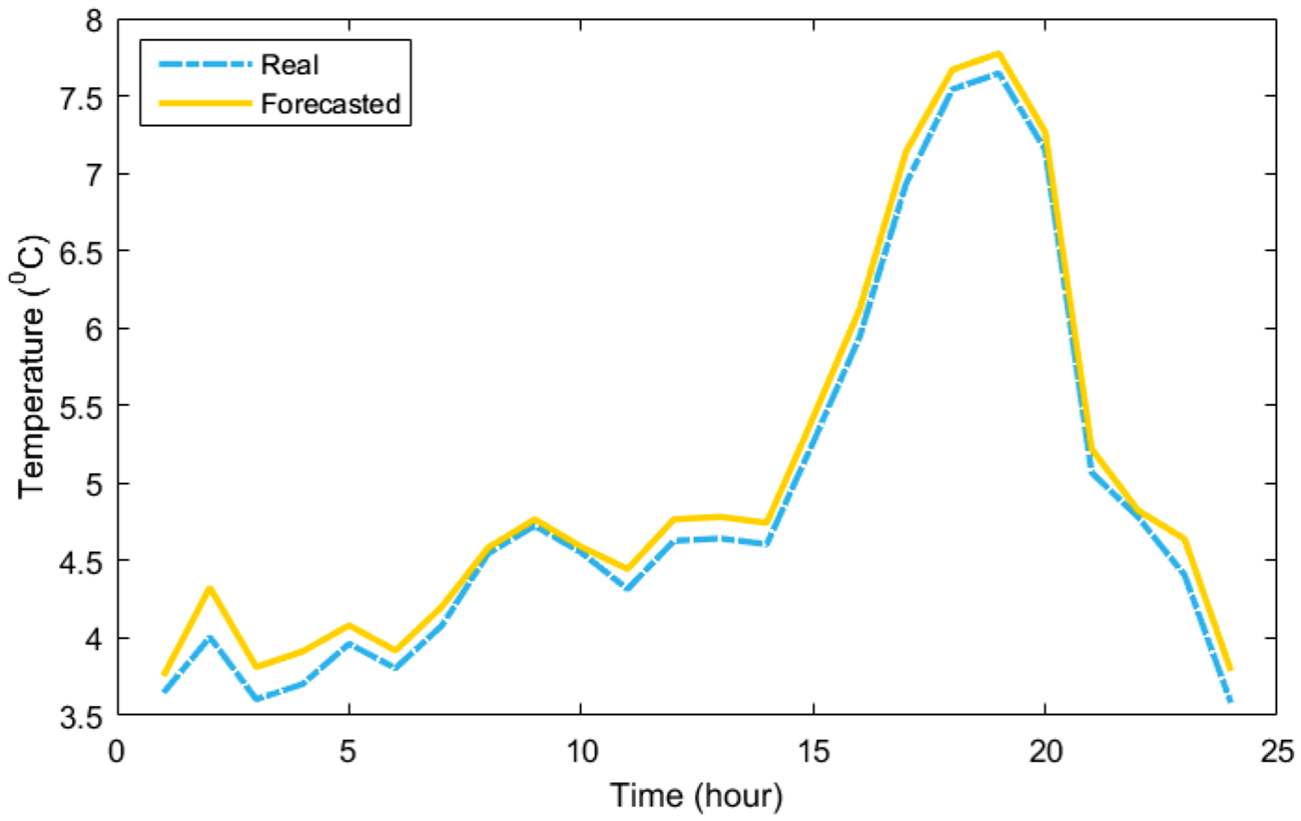
The real and forecasted temperature during a day.

**Figure 9:**
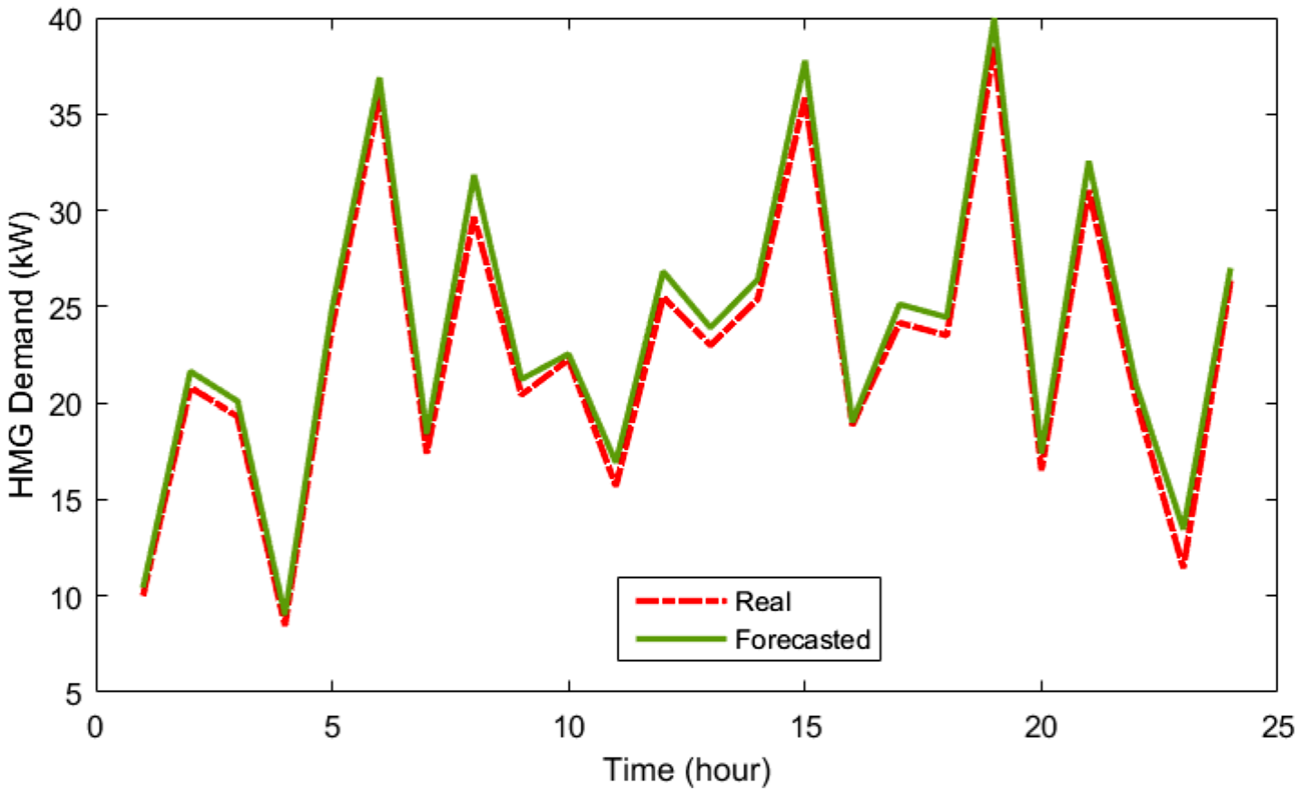
The real and forecasted HMG load during a day.

Table 3 provides the MLP-ANN's starting parameters such as training algorithm (TA), MSE, Epoch, validation checks (VCH), input layers number (ILN), hidden layers number (HLN), output layers number (OLN), and transfer function (TF). Table 4 displays the outcomes of the MLP-ANN prediction for load and meteorological data. As can be demonstrated based on their recorded daily data, which are displayed in Figs. 6, 7, 8 and 9, respectively, the MLP-ANN forecasts irradiance, wind speed, ambient temperature, and HMG demand values. This section presents the findings via the MOIKOA of the energy system in the network. The actual meteorological data is derived from Refs.^10,13^.

**Table 3:** MLP-ANN's initial configuration parameters.

Item	Description
TA	Levenberg–marquardt
MSE	0
Epoch	1500 iterations
VCH	50
ILN	1
HLN	1
OLN	1
TF	Tansig and purelin

**Table 4:** The MLP-ANN's predictions for demand data and meteorology.

Item	MSE		RMSE		R	
	Training	Testing	Training	Testing	Training	Testing
SI	0.0020	0.0018	0.0445	0.0434	0.9915	0.9916
AT	4.245	3.3712	2.0603	1.9316	0.9760	0.9767
WS	4.7137	4.4873	2.1711	2.1183	0.2300	0.2350
LD	0.2013	0.2136	0.4486	0.4621	0.9937	0.9939

### Results of scenario#1

**Figure 10:**
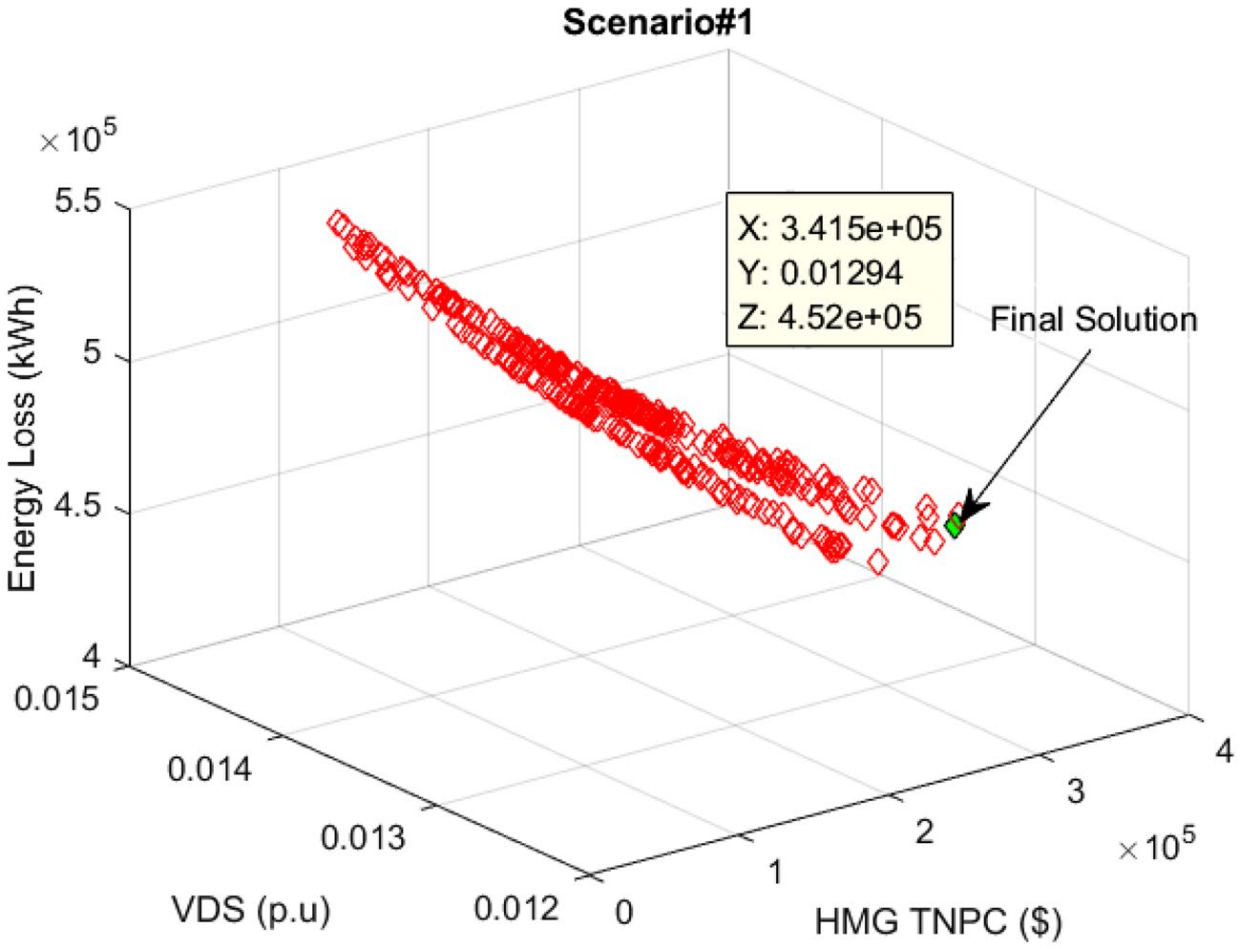
Pareto optimal solution set using the MOIKOA for Scenario#1.

The outcomes of multi-objective optimizing the PV/WT/BES microgrid in the network are presented via a new MOIKOA algorithm and the FDMT to achieve the best interactive solution considering meteorological data and real load demand as scenario 1. The Pareto optimal solution set curve for scenario 1 is obtained using the MOIKOA algorithm in Fig. 10. Based on the FDMT process, between the optimal solutions, the final solution (in green color) has been determined and the values of each of the goals of annual power losses, the value of network voltage oscillations and the cost of purchasing power from HMG corresponding to the final solution are given in Table 1.

As can be seen in Table 5, the capability of MOIKOA is evaluated with other multi-objective methods including MOKOA, MOPSO and MOGA. It should be noted that each algorithm is implemented independently 30 times and for each algorithm, the population number and maximum iteration are selected to be 50 and 100, respectively. The results showed that contrasted to other multi-objective approaches, the proposed MOIKOA achieved lower annual power losses, network voltage deviations, and HMG power costs. The MOIKOA has installed 470 kW of PV power, 817 kW of wind power, and 1244 kWh of battery capacity in Bus 17 of the network. Therefore, the amount of annual losses is reduced from 666,052 kW in the basic state to 423,227 kW and the voltage oscillations is declined from 0.0147 p.u to 0.0129 p.u and $340,630 cost of power purchased from HMG has been obtained.

**Table 5:** Results of fuzzy multi-objective optimization of a hybrid PV/WT/BES microgrid in 33-bus network (Scenario#1).

Item	Base net	MOIKOA	MOKOA	MOPSO	MOGA
Number of PV	–	470	606	463	345
Number of WT	–	817	819	801	818
Number of BES	–	1244	1006	1442	1154
Location of HMG	–	Bus 17	Bus 16	Bus 16	Bus 15
Annual energy loss (kW)	666,052	423,227	423,519	423,873	424,106
Voltage deviation (p.u)	0.0147	0.0129	0.0130	0.0130	0.0131
HMG purchased power cost	–	340,630	344,517	342,362	342,439

**Figure 11:**
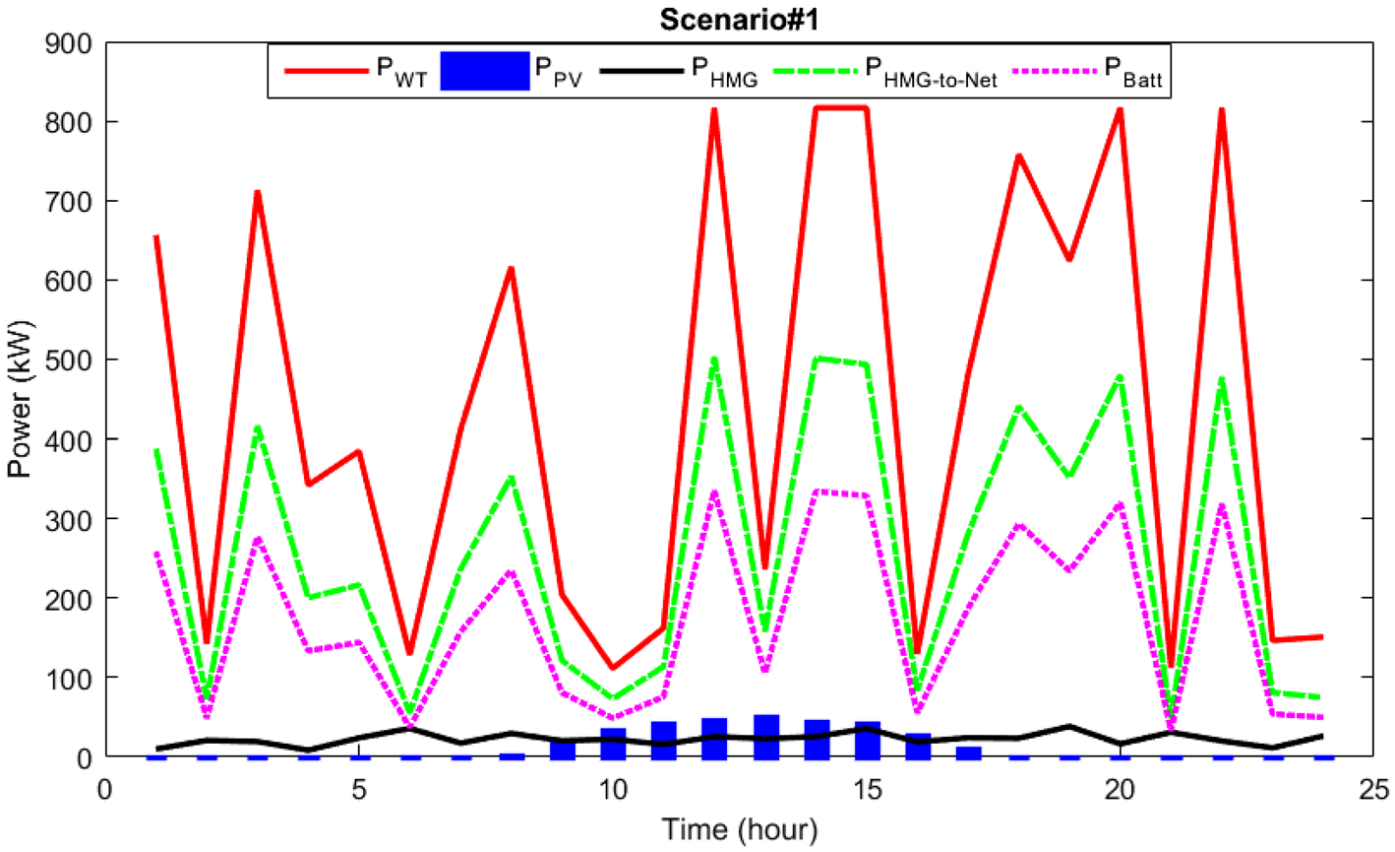
Power dispatch using the MOIKOA for Scenario#1.

Figure 11, shows the power dispatch of different HMG equipment along with microgrid load demand. It can be seen that wind and PV source are the sources of power generation, and the extra power required by the HMG load is divided into two parts. Based on the considered modeling, it is considered that 40% of the excess power will be transferred into the batteries for energy storage to supply the HMG load, and the rest (60%) will be sold to the network. Therefore, integrated energy production resources with battery reserve management have provided the possibility of continuous supply of microgrid load, then by injecting planned power into the network, they have also provided the conditions for improving the network performance.

**Figure 12:**
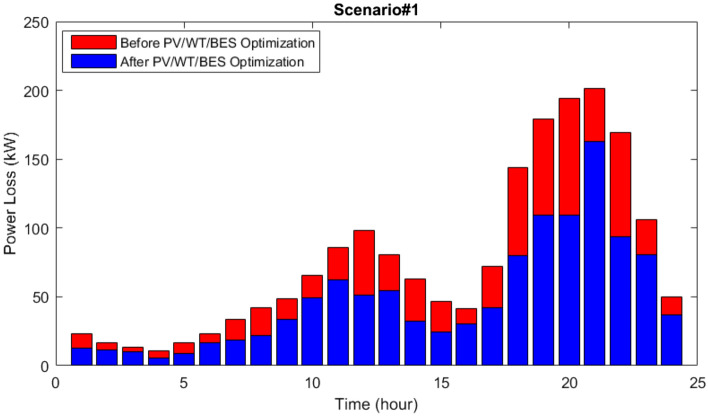
Power losses of the 33-bus network via the MOIKOA for Scenario#1.

**Figure 13:**
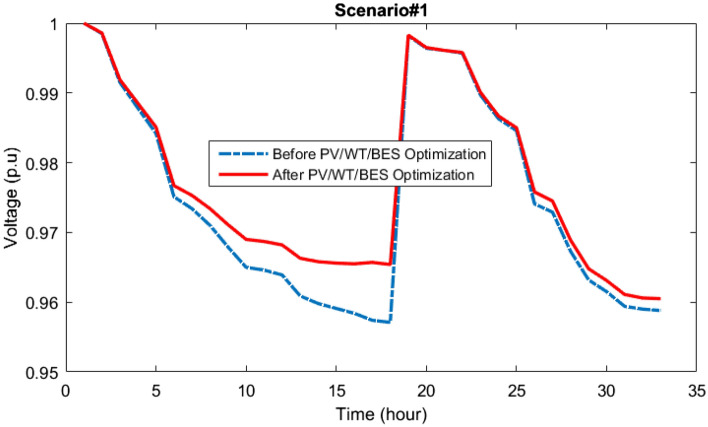
Voltage deviations of the 33-bus network via the MOIKOA for Scenario#1.

In Figs. 12 and 13, curve of network lines active losses along with network buses voltage oscillations are shown. As it can be seen, the microgrid optimization in the network to compute the optimum location and size of the equipment has decreased losses and also enhanced its voltage profile.

This section presents an equal comparison of the numerical results derived from the MOIKOA, MOKOA, MOPSO, and MOGA methods, taking into account 30 separate runs. The outcome of each method has been compared using the C index (CI) since multi-objective optimization algorithms possess a more sophisticated search engine performance than single-objective methods^42^. Two algorithms' abilities to solve the multi-objective optimization issue have been contrasted using the C index. It is presupposed in the aforementioned criteria that *S*_1_ and *S*_2_ are the two algorithms' output PSS set. (*S*_1_, *S*_2_) is explained according to^42^ and represents the percentage of answers in set *S*_2_ that are significantly outnumbered by answers in set *S*_1_.

63 $$C(S_{1} ,S_{2} ) = \frac{{\left\{ {\left. {s_{2} \in S_{2} ;\,\exists s_{1} \in S_{1} \,\,:\,\,s_{1} \le s_{2} } \right\}} \right.}}{{\left| {S_{2} } \right|}} \times 100$$

where *s*_1_, *s*_2_ are, respectively, the PSSs in sets *S*_1_, *S*_2_. A better PSS is indicated by a solution with a greater C index, which is calculated as the average of n separate executions.

Table 6 displays the C indexes for different algorithm types. The outcomes demonstrated that, on average, 86.74%, 63.23%, and 70.54% of the answers provided by the MOIKOA outweigh those obtained by MOKOA, MOPSO, and MOGA in rows 2, 4, and 6. Additionally, in contrast to the MOKOA, MOPSO, and MOGA approaches, the superior capability of the MOIMRFO in achieving better PSSs is demonstrated.

**Table 6:** Comparing various algorithms with the CI for scenario#1.

CI	Mean	STD	Maximum	Minimum
C(MOIKOA, MOKOA)	13.56	18.20	37.65	18.44
C(MOKOA, MOIKOA)	86.74	15.41	100	0
C(MOIKOA, MOPSO)	41.09	32.16	68.85	20.27
C(MOPSO, MOIKOA)	63.23	26.32	84.05	6.94
C(MOIKOA, MOGA)	37.25	26.02	56.43	18.47
C(MOGA, MOIKOA)	70.54	22.97	84.38	16.06

### Results of scenario#2

**Figure 14:**
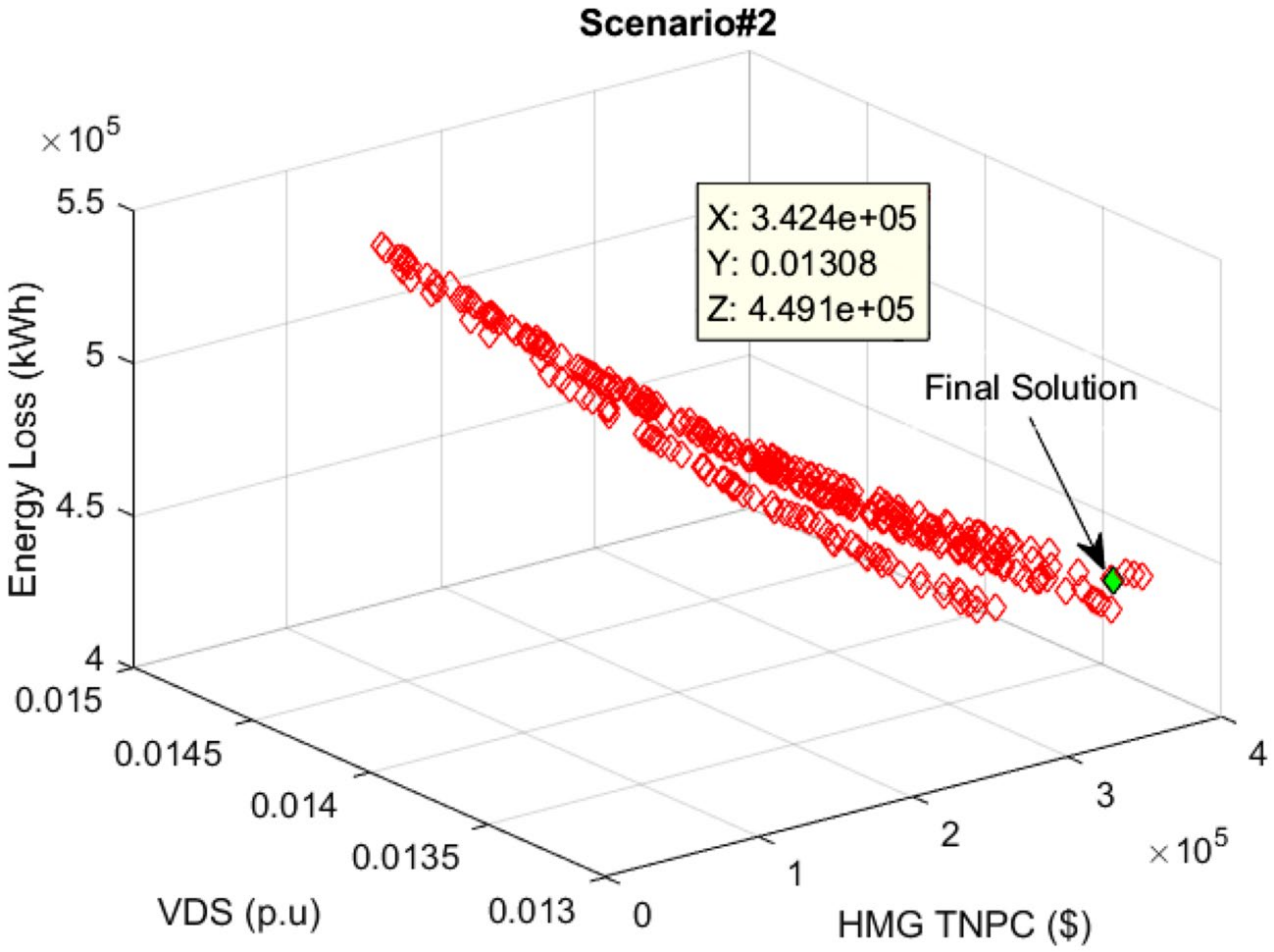
Pareto optimal solution set using the MOIKOA for Scenario#2.

This section presents the findings from the multi-objective optimization of the PV/WT/BES microgrid in the network through the new MOIKOA and the FDMT. The goal was to find the best interactive solution taking into account the load demand predicted by the machine learning algorithm as scenario 2 and meteorological data. Figure 14 shows the PSS set for scenario 2 via the MOIKOA method. Table 7 provides the values of each goal of annual power losses, the voltage oscillations, and the purchasing power cost from HMG that corresponds to the final solution. The final solution (in green) was selected among the optimal solutions depending on the FDMT process.

**Table 7:** Results of fuzzy multi-objective optimizing the PV/WT/BES system using the MOIKOA in 33-bus network (Scenario#2).

Item	Base net	MOIKOA	MOKOA	MOPSO	MOGA
Number of PV	–	234	254	277	484
Number of WT	–	892	902	881	855
Number of BES	–	1613	1248	1190	880
Location of HMG	–	Bus 17	Bus 16	Bus 16	Bus 18
Annual energy loss (kW)	666,052	438,135	440,637	441,550	426,819
Voltage deviation (p.u)	0.0147	0.0132	0.0133	0.0133	0.0133
HMG purchased power cost	–	347,365	347,735	347,880	349,417

Table 7 illustrates how the MOIKOA performance in scenario 2 is contrasted with MOKOA, MOPSO, and MOGA. It should be mentioned that every algorithm is run independently thirty times, with 50 and 100 repetitions being the maximum for each algorithm, respectively. The findings demonstrated that the suggested MOIKOA produced lower values of yearly power losses, network voltage variations, and HMG power cost in comparison to previous multi-objective techniques. In Bus 17 of the network, the MOIKOA has installed 234 kW of PV power, 892 kW of wind power, and 1,613 kWh of battery capacity. As a result, the base network's yearly losses are decreased from 666,052 to 438,135 kW, voltage variations are cut from 0.0147 to 0.0132 p.u., and $347,365 worth of power was acquired from HMG.

**Figure 15:**
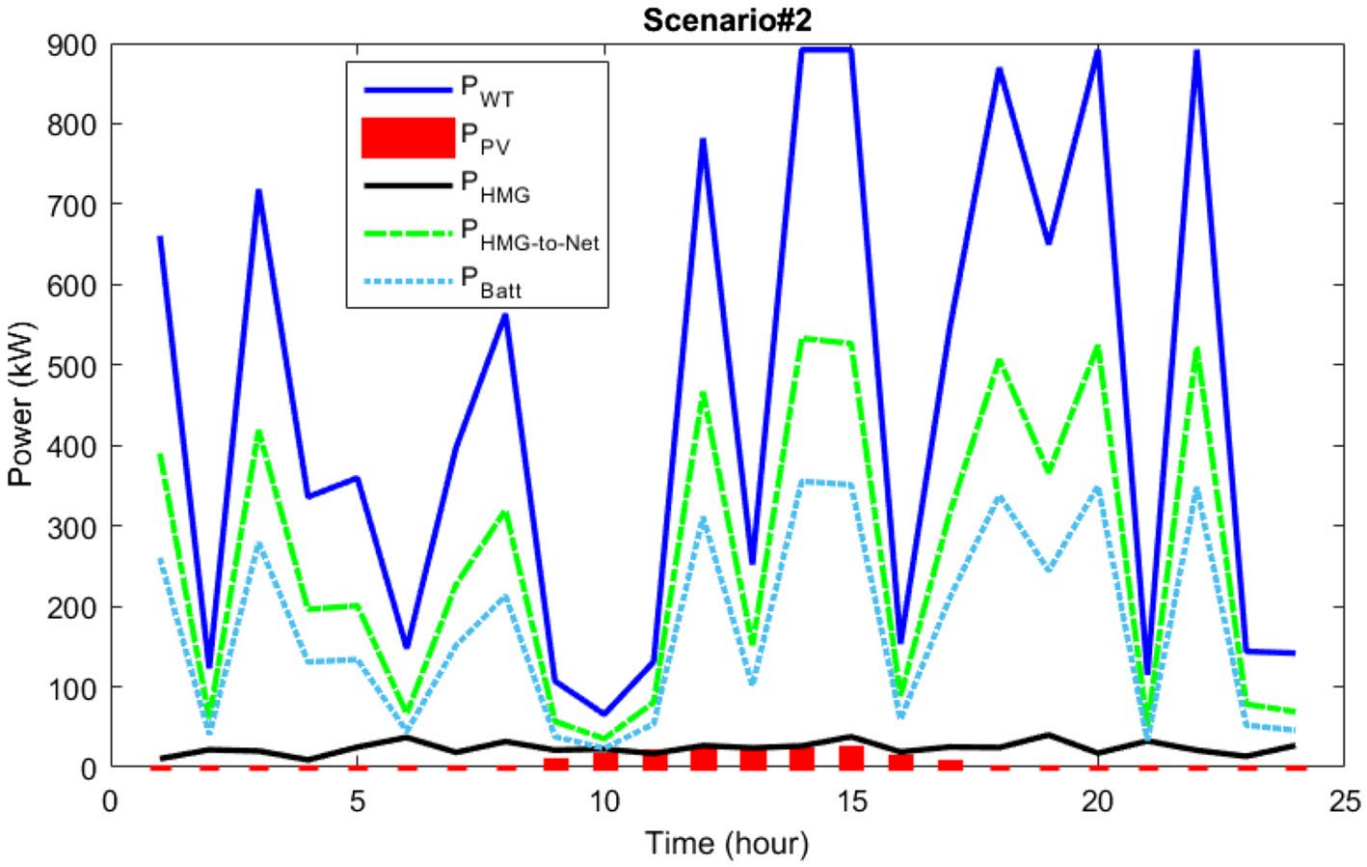
Power dispatch via the MOIKOA for Scenario#2.

In scenario 2, the power dispatch of various microgrid components and microgrid load demand are demonstrated in Fig. 15. As it is observed, the primary power generating sources are solar and photovoltaic (PV) systems, and the surplus power needed by the hybrid generation load is split into two halves. According to the modeling under consideration, it is anticipated that 60% of the extra power will be sold to the network and 40% will be injected into the batteries for energy storage to satisfy the HMG load. As a result, integrated energy-generating sources with battery reserve management have made it possible for microgrid loads to be supplied continuously. They have also made it possible for the grid to function better by introducing programmed power into the network.

**Figure 16:**
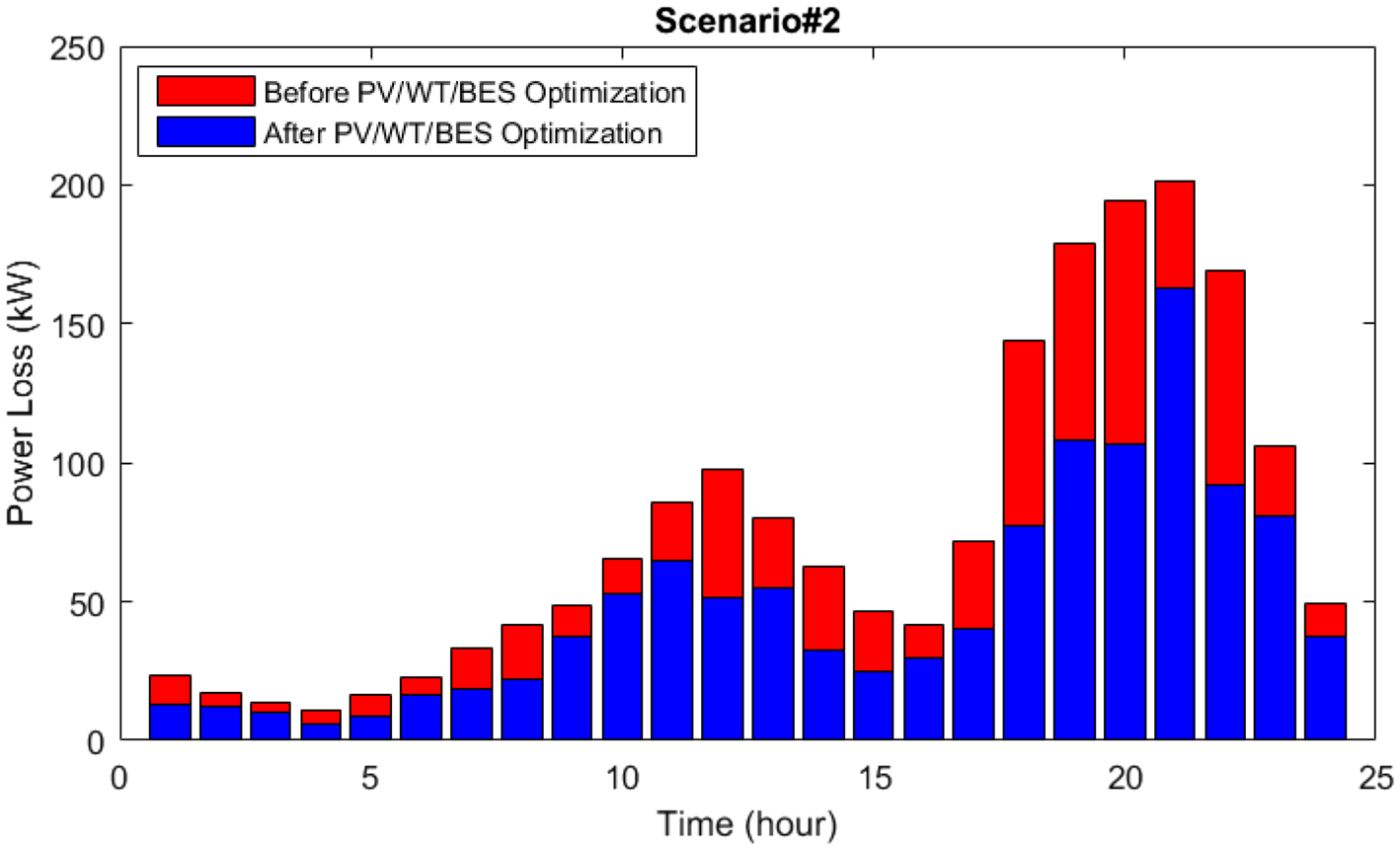
The losses of the 33-bus network via the MOIKOA for Scenario#2.

**Figure 17:**
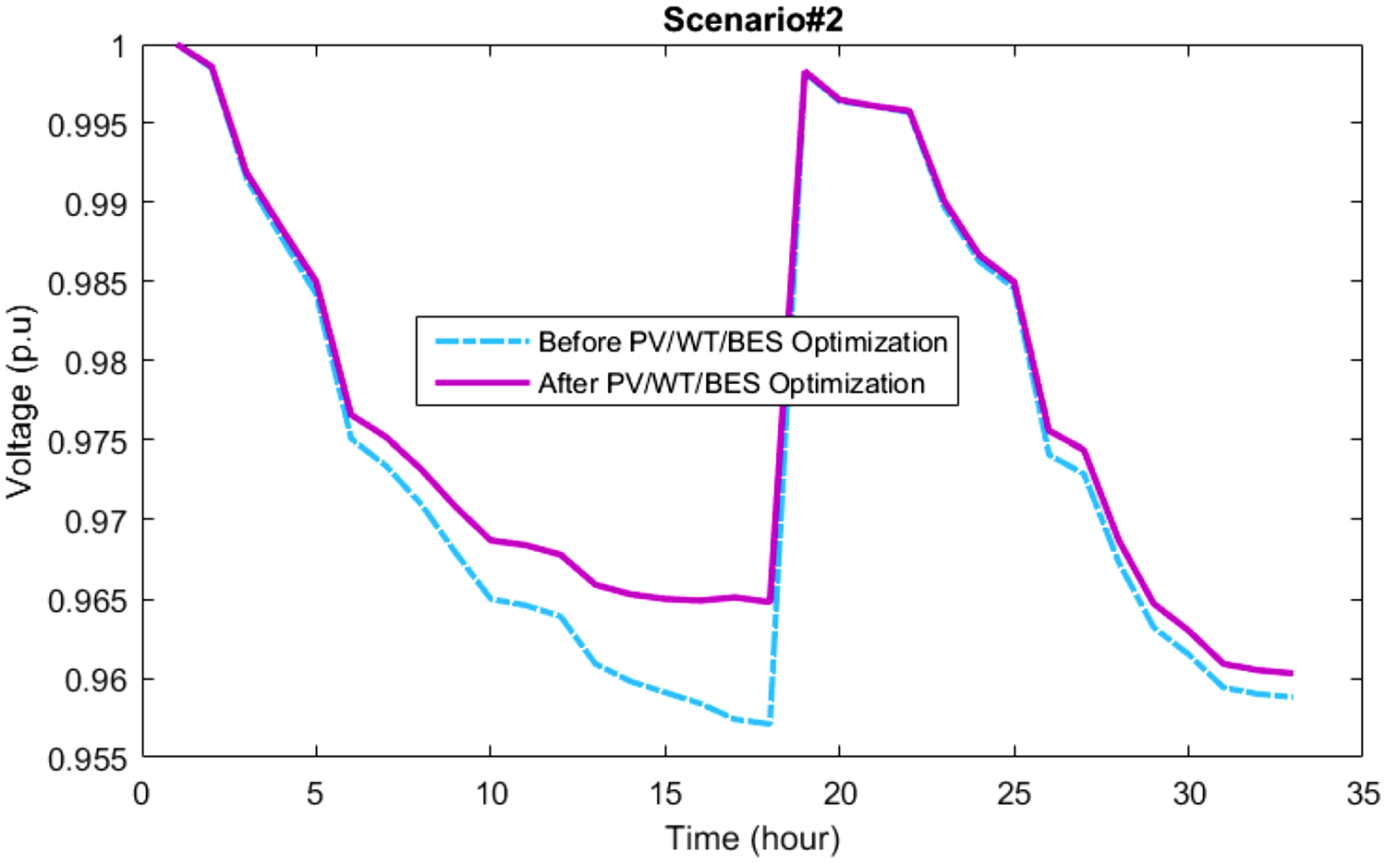
The voltage profile of the 33-bus network via the MOIKOA for Scenario#2.

The active loss curves of the network lines and the voltage variations for scenario 2 are depicted in Figs. 16 and 17. As can be observed, the voltage profile is improved and network losses have been decreased as a result of the energy microgrid's optimization through the selection of the best installation site and equipment capacity.

Table 8 displays the C indices for several methods. The findings demonstrated that, on average, 83.25%, 76.04%, and 80.29% of the answers acquired by the MOIKOA outweigh those provided by MOKOA, MOPSO, and MOGA in rows 2, 4, and 6. Additionally, in contrast to the MOKOA, MOPSO, and MOGA approaches, the higher capacity of the MOIKOA to achieve a better Pareto solution set is demonstrated.

**Table 8:** Comparing various algorithms with the CI for scenario#2.

CI	Mean	STD	Maximum	Minimum
C(MOIKOA, MOKOA)	10.12	32.38	29.45	20.19
C(MOKOA, MOIKOA)	83.25	18.94	100	0
C(MOIKOA, MOPSO)	47.62	29.67	66.13	22.74
C(MOPSO, MOIKOA)	76.04	21.40	81.34	11.66
C(MOIKOA, MOGA)	29.11	28.54	49.71	20.32
C(MOGA, MOIKOA)	80.29	16.05	72.36	14.63

### The scenarios results comparison

This section compares the performance of the suggested methodology in two scenarios, 1 and 2, which are based on actual and predicted data, respectively. The MOIKOA algorithm is used to find the ideal capacity of the HMG components and the installation placement of the HMG in the network using the fuzzy multi-objective optimization problem. As far as we are aware, using anticipated data for solving the microgrid optimization problem in the network is a more accurate method of optimizing the system for the day ahead of schedule than using actual or estimated data. Table 9 shows that, in scenario 2, the PV power has decreased from 470 to 234 kW. This decrease is due, in part, to a decrease in radiation intensity based on the data that was expected. Due to variations in the wind speed profile and the non-linearity of wind power generation according to wind speed, scenario 2's wind power has also increased when compared to scenario 1. In contrast, scenario 2's battery storage capacity has been raised to improve network performance under these circumstances by lowering the power set of renewable resources relative to scenario 1. As compared to scenario 1 using actual data, the results indicated that taking into account the anticipated data in the microgrid optimization problem in the network (situation 2) raised the yearly cost of losses from $423,227 to $438,135. Additionally, the cost of the electricity that was purchased from HMG went from $340,630 to $347,365, and the amount of network bus voltage variation increased from 0.0129 to 0.0132 p.u. Consequently, without considering the comprehensive forecasted data, the optimization and detailed planning of storage-based hybrid microgrids fail to inform the network planning of the logical capacities of storage to enhance the network's performance by better compensating for fluctuations in renewable energy sources' power. Table 9 shows that when the microgrid multi-objective optimization problem is implemented, the annual power loss cost, voltage oscillations, and power purchase from the HMG increase by 3.50%, 2.33%, and 1.98%, respectively when the forecasted data based on the machine learning algorithm is compared to the real data.

**Table 9:** Results comparison of scenarios 1 and 2.

Item	Scenario#1	Scenario#2	Deviation value
Number of PV	470	234	Considerable reduction of PV power
Number of WT	817	892	Increasing the WT power
Number of BES	1244	1613	Considerable increasing the battery energy storage
Location of HMG	Bus 17	Bus 17	Similar location
Annual energy loss (kW)	423,227	438,135	3.5% increasing the energy losses
Voltage deviation (p.u)	0.0129	0.0132	2.33% increasing the voltage deviations
HMG purchased power cost	340,630	347,365	1.98 increasing the HMG power cost

**Figure 18:**
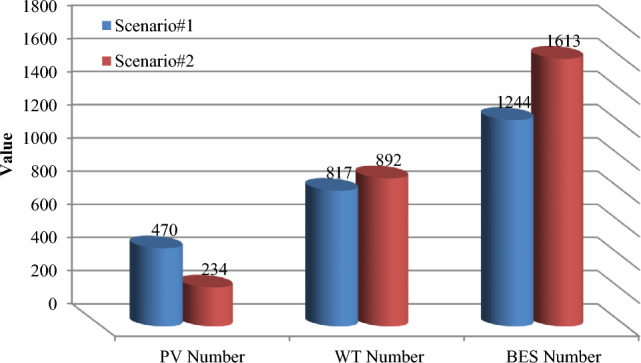
Comparison of the best solution in scenarios 1 and 2.

**Figure 19:**
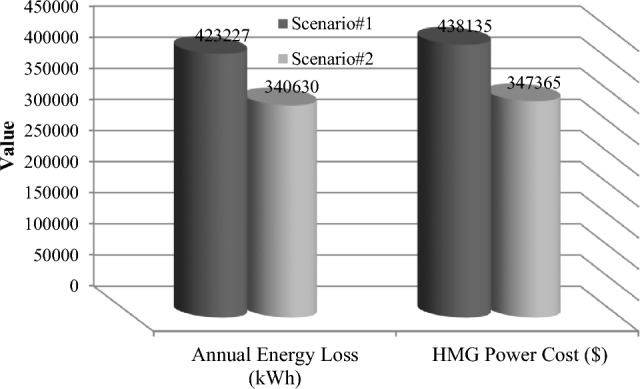
Comparison of the annual energy loss and HMG purchased power cost in scenarios 1 and 2.

**Figure 20:**
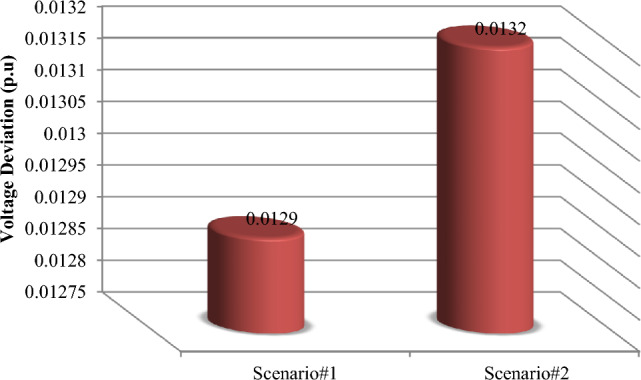
Comparison of the network voltage deviation in scenarios 1 and 2.

The results of various scenarios are shown in Figs. 18, 19 and 20. These results include the set of optimal solutions (Fig. 18), the annual losses cost and the cost of buying power from HMG (Fig. 19), and the voltage oscillations of the 33-base network (Fig. 20) in scenarios 1 and 2. It is evident from these results that taking into account the machine learning algorithm's forecasted data instead of the real data has increased the annual losses cost, the cost of buying power from HMG, and voltage deviations. While using predicted data gives network planners a true and accurate optimization structure that gives them the ability to make informed judgments, increasing these values does not necessarily indicate bad data performance.

### Results of DOD variations effect

The depth of discharge (DOD) refers to the amount of battery charge used relative to its total capacity, expressed as a percentage. It is calculated by dividing the remaining battery charge by the battery's nominal capacity. This section examines how variations in DOD influence the microgrid's ability to enhance network features such as energy losses and voltage oscillations, as well as the battery storage system's contribution and the cost of purchasing power from the microgrid. As shown in Table 10, an increase in DOD percentage, from 10 to 80%, leads to a reduction in battery energy capacity but allows the battery to provide more energy to the hybrid microgrid (HMG) to compensate for load power loss. Table 10, indicates that this rise in DOD percentage results in decreased storage capacity and resource generation. The findings also reveal that higher DOD levels correlate with reduced annual loss cost, voltage oscillations, and power purchasing costs from the microgrid, due to improved storage participation and better energy management. Specifically, increasing DOD to 80% improves the microgrid's efficiency by achieving network goals. When DOD is adjusted from 10 to 80%, annual losses decrease to 423,868 kW, voltage variation drops to 0.01300 p.u. from 0.01334 p.u., and the cost of electricity purchased from HMG decreases from $388,190 to $343,388. Thus, a higher DOD enhances network performance and increases battery participation.

**Table 10:** Results of fuzzy multi-objective optimization of the PV/WT/BES system in 33-bus network (Scenario#3).

Item	10%	20%	30%	40%	50%	60%	70%	80%
Number of PV	365	330	313	293	279	266	246	234
Number of WT	997	993	984	978	960	935	912	892
Number of BES	1986	1971	1946	1912	1883	1852	1809	1613
Location of HMG	Bus 17	Bus 17	Bus 17	Bus 17	Bus 17	Bus 17	Bus 17	Bus 17
Annual energy loss (kW)	461,730	457,510	453,183	446,082	441,863	434,932	428,025	423,868
Voltage deviation (p.u)	0.01334	0.01330	0.01326	0.01319	0.01314	0.01309	0.01304	0.01300
HMG purchased power cost	388,190	379,022	372,866	363,214	356,769	350,448	348,297	343,388

**Figure 21:**
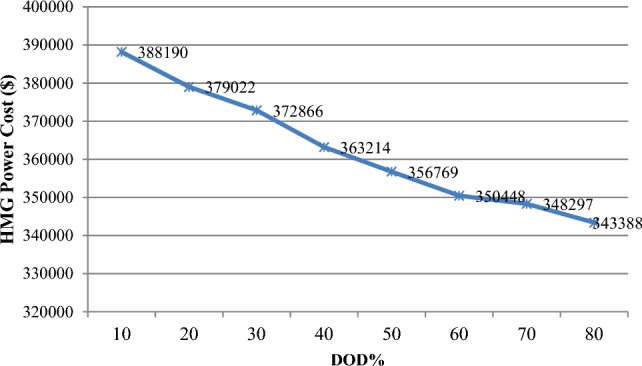
The HMG power cost variations versus DOD%.

**Figure 22:**
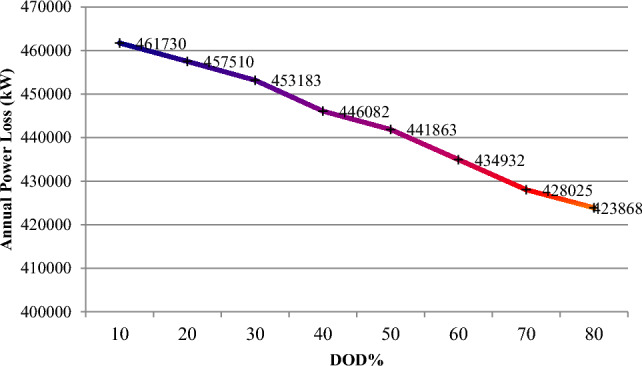
The annual power loss variations versus DOD%.

**Figure 23:**
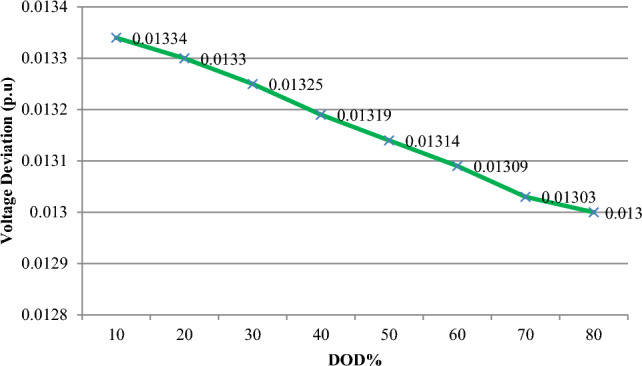
The voltage deviation variations versus DOD%.

Figure 21 shows that as the percentage of DOD increases, the network's cost of acquiring power from HMG has decreased; Fig. 22 shows that the annual power loss has also decreased; and Fig. 23 shows that the amount of voltage deviations in the 33-bus network has increased along with the DOD percentage.

## Conclusion

In this study, a multi-objective structure for a PV/WT/BES microgrid optimization in a 33-bus network was implemented for minimizing the annual energy losses, to minimize the network bus voltage oscillations, and minimize the cost of purchasing power from the microgrid by the network. The problem is implemented in three scenarios. First, in scenario 1, the optimization problem was done with real data, and the variables, i.e., the position of the microgrid and the optimal size of PV and wind resources along with battery storage capacity, were determined using a new improved multi-objective algorithm named MOIKOA. Then, in scenario 2, the optimization problem was done considering the forecasted data based on the presented machine learning algorithm. Then, in scenario 3, the effect of battery DOD changes has been evaluated as the important contribution of storage devices in the HMG optimization in the network in improving each of the network objectives. The study outcomes are listed as follows:

- The findings presented that the suggested multi-objective method was easily capable of computing the final interactive answer between the set of answers in such a way that a favorable compromise between different objectives is achieved. Also, the optimal variables are found and the best microgrid capability with more effectiveness is extracted in improving the network characteristics.
- Results of scenario 1 considering real data showed that the proposed multi-objective framework based on the MOIKOA achieved lower annual power losses, network voltage deviations, and HMG power cost compared to the other multi-objective algorithms. Based on the proposed methodology in scenario 1, the annual losses were decreased from 666,052 to 423,227 kW, voltage deviations were decreased from 0.0147 to 0.0129 p.u and the HMG power cost was obtained to $340,630.
- The MLP-ANN predicts irradiance, wind speed, ambient temperature, and HMG demand data according to the recorded daily data, and scenario 2 was implemented considering the forecasted data. The results of scenario 2 considering forecasted data showed that the microgrid multi-objective optimization problem in this scenario causes an increase of 3.50%, 2.33%, and 1.98%, respectively, in the annual cost of losses, voltage deviations, and the cost of purchasing power from the HMG compared to the real data-based optimization.
- The effect of DOD variations was investigated in scenario#3 and the findings cleared that by increasing the DOD% from 10 to 80%, the annual energy losses were decreased by 8.20%, voltage deviation was declined by 2.55% reduction, and the HMG power cost was decreased by 11.54%. Therefore, a high percentage of DOD has increased battery participation and improved network objectives.
- Robust multi-objective optimizing the PV/WT microgrid system incorporating multi-energy storage is suggested for future work using information gap decision theory considering efficiency, and reliability of hybrid microgrids and incorporating the adaptive real-time optimization.

## Data Availability

The datasets used and/or analyzed in the study available from the corresponding author for reasonable request.
